# Prophage induction drives soybean rhizobacterial community differentiation and nutrient cycling benefiting root development

**DOI:** 10.1093/ismeco/ycaf203

**Published:** 2025-11-06

**Authors:** Yujun Zhong, Yingyue Zhang, José Luis López Arcondo, Ruoyi Xu, Mark Radosevich, Jeffery L Dangl, Bas E Dutilh, Xiaolong Liang

**Affiliations:** CAS Key Laboratory of Forest Ecology and Silviculture, Institute of Applied Ecology, Chinese Academy of Sciences, No. 72 Wenhua Road, Shenyang, Liaoning Province 110016, China; University of Chinese Academy of Sciences, Beijing 101408, China; CAS Key Laboratory of Forest Ecology and Silviculture, Institute of Applied Ecology, Chinese Academy of Sciences, No. 72 Wenhua Road, Shenyang, Liaoning Province 110016, China; Institute of Carbon Neutrality Technology and Policy, Shenyang University, Shenyang 110044, China; Institute of Biodiversity, Faculty of Biological Sciences, Cluster of Excellence Balance of the Microverse, Friedrich Schiller University Jena, Jena 07743, Germany; Instituto Andino Patagónico de Tecnologías Biológicas y Geoambientales, Bariloche, Rio Negro 8400, Argentina; CAS Key Laboratory of Forest Ecology and Silviculture, Institute of Applied Ecology, Chinese Academy of Sciences, No. 72 Wenhua Road, Shenyang, Liaoning Province 110016, China; University of Chinese Academy of Sciences, Beijing 101408, China; Department of Biosystems Engineering and Soil Science, The University of Tennessee, Knoxville, TN 37996, United States; Department of Biology, University of North Carolina at Chapel Hill, Chapel Hill, NC 27599, United States; Howard Hughes Medical Institute, University of North Carolina at Chapel Hill, Chapel Hill, NC 27599, United States; Institute of Biodiversity, Faculty of Biological Sciences, Cluster of Excellence Balance of the Microverse, Friedrich Schiller University Jena, Jena 07743, Germany; Theoretical Biology and Bioinformatics, Department of Biology, Science for Life, Utrecht University, Utrecht 3584 CH, The Netherlands; CAS Key Laboratory of Forest Ecology and Silviculture, Institute of Applied Ecology, Chinese Academy of Sciences, No. 72 Wenhua Road, Shenyang, Liaoning Province 110016, China; University of Chinese Academy of Sciences, Beijing 101408, China; Howard Hughes Medical Institute, Department of Biology, University of North Carolina at Chapel Hill, Chapel Hill, NC 27599, United States

**Keywords:** viral lysis, viral shunt, rhizosphere, virome, soil, interaction network

## Abstract

Bacteriophages, lytic or lysogenic, play critical roles in structuring different soil bacteriomes and driving their functionality. Lysogeny is favored in the plant rhizosphere and may play a major role in plant–rhizobacteria assembly and function. However, the ecological footprint and consequence of prophage activity in the rhizosphere are poorly understood. Here, we conducted a 35-day pot experiment to examine how prophage induction influences soybean rhizosphere viromes and bacterial communities, along with associated changes in nutrient cycling and plant development. The results showed that mitomycin C-induced prophage induction triggered immense viral production, altering virome structure—with more observed species richness in the rhizosphere. We observed a greater impact on the rhizosphere virome than on the bulk soil virome. The resulting lysis decreased the soil organic matter content but significantly increased dissolved organic carbon and nitrate contents in the soil, which improved soil nutrient conditions and stimulated soybean root development. Prophage induction markedly influenced the rhizobacterial community structure, resulting in reduced community diversity. The enrichment of fast-growing bacterial populations was stimulated, suggesting that viral lysis increased microbial activities and accelerated nutrient turnover. The bacterial interaction network was drastically shifted, with complexity being decreased in the bulk soil and increased in the rhizosphere, potentially stimulating the differentiation of the bacterial communities. Together, our results demonstrated that induction of prophages can cause extensive nutrient turnover and variations in plant–rhizobacteria interactions, driving the rhizobacterial community assembly process. This study provides novel insights into the mechanisms of phages controlling microbial function in primary production and soil carbon storage by modulating microbial traits (e.g., carbon use efficiency, growth rate, death, and community assembly) and via processes like the viral shunt.

## Introduction

Bacterial populations in natural environments are frequently exposed to bacteriophage (phage) infection. Phage-induced lysis is a major driver of bacterial diversity and metabolic activity [1, 2]. Large proportions of bacterial populations within an environment can be impacted by lytic phages, leading to accelerated biomass turnover. Antagonistic interactions promote evolutionary changes in both phage and host communities and are directly linked to many important ecological processes such as biogeochemical cycling and plant–microbial interactions [3, 4]. Besides as extracellular particles in the environment, phages may exist in a lysogenic state, where their genome is stably integrated into that of their bacterial host. Prophages function as gene transfer agents and serve as a primary source of genetic diversity in host communities. Prophage-carried genes may also be directly involved in bacterial metabolism [5].

Lysogeny is common in soil bacterial populations. Under certain environmental or biological conditions, prophages integrated in bacterial genomes can become active and enter the lytic cycle, a process known as prophage induction. For example, the DNA synthesis–inhibiting agent and genome stress-inducing agent mitomycin C has been widely used as a chemical reagent for prophage induction in the laboratory to study the lysogenic phages and host populations in natural or laboratory environments [6–8]. Prophages maintained in bacterial genomes are pervasive in nature, and the lysogeny-to-lysis switch is very common, exerting a direct influence on host community development and function [9]. Phage reproduction rates and strategies reflect complex phage–host dynamics and are associated with many environmental and biotic factors, but the impact of dynamic interactions remains under-investigated. Understanding how lysogeny modulates microbial traits in the rhizosphere is essential for interpreting its ecological implications in plant-associated soil environments.

Soil is teeming with abundant life and high biodiversity and supports biogeochemical cycles and food production [10]. The complex bio- and geo-chemical processes in soil are of importance for ecosystem services. The rhizosphere is the plant root–soil interface and contains a higher biomass with high levels of diverse organic carbon secreted by plant roots. These secretions provide nutrients and signaling molecules that drive a continuum of associated soil-derived microorganisms [11]. Thus, the rhizosphere sustains active microbial and cross-kingdom interactions between plants and microbes. Rhizosphere-resident microbial populations and processes can influence host plant growth and fitness [12, 13]. While the rhizosphere has a higher bacterial density than bulk soil, phage densities are not significantly different between the two, leading to a notable overall decrease in the phage-to-bacteria ratios [14].

Within rhizobacterial cells, many phages persist as prophages integrated in host genomes, and previous studies have shown that lysogeny is promoted under relatively high bacterial densities [15]. Lysogeny may benefit host bacterial cell fitness and stimulate relationships between rhizobacterial populations and plants. Prophage induction may also significantly affect rhizosphere microbial processes, because lysis/lysogeny decisions can be closely associated with host growth rate, physiological state, and multiplicity of infection [1]. However, the ecological footprint and function of prophages in the rhizosphere are poorly understood. In particular, little is known about how bacterial populations are impacted by prophage induction in either the rhizosphere or in bulk soils.

We sought to assess the effects of prophage induction on the differentiation of bacterial community assembly processes in the rhizosphere and the bulk soil. We also evaluated how the lysogeny–lysis switch affects nutrient cycling and its feedback on bacterial community structure. Specifically, we analyzed the impacts of prophage induction on soil nutrient conditions, virome composition, bacterial community assembly processes, and soybean root development. We hypothesized that prophage induction might cause extensive nutrient turnover and variations in plant–rhizobacteria interactions, and that the viral-induced events may markedly influence bacterial community succession and rhizosphere bacteriome differentiation from bulk soil.

## Materials and methods

### Soil sampling and cultivation

**Figure 1:**
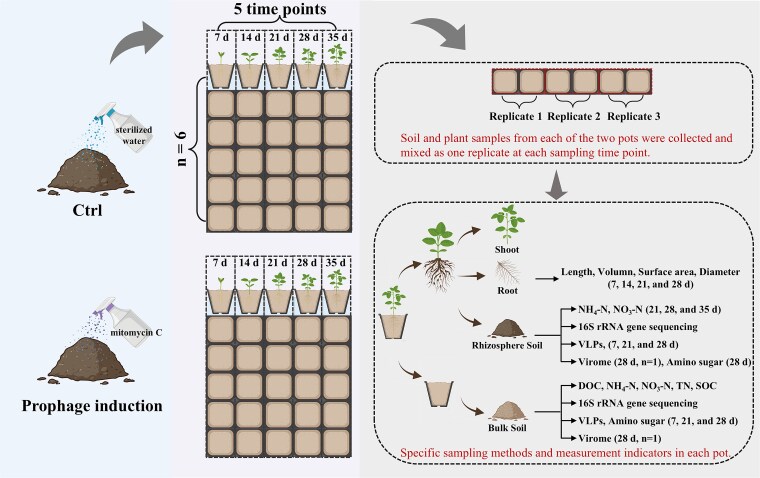
Schematic overview of the experimental design and sample processing workflow. Soybean plants were grown in soil under two treatments: Prophage induction (initial soil moisture adjusted to 60% at experiment start with mitomycin C to a final concentration of 0.5 μg·g^−1^ dry soil) and control (applied sterile deionized water to adjust the initial soil moisture). Destructive sampling was conducted at five timepoints (Days 7, 14, 21, 28, and 35), with six biological replicates per treatment per timepoint. Rhizosphere soil, bulk soil, root, and shoot samples were collected for microbial, physicochemical, and phenotypic analyses. 16S rRNA gene amplicon sequencing was performed on 60 soil samples. VLP abundance was quantified over time. Virome sequencing was conducted on pooled soil samples (three replicates per group) from Day 28, including rhizosphere and bulk compartments under both treatments. Figure 1 was created by BioRender.com.

Soil samples of the upper soil layer (0–20 cm) were collected from an agricultural field site for maize production located at the Shenyang Agroecosystem Research Station (41°31′ N, 123°24′ E). Soils were thoroughly mixed and stored in sterilized bags. Upon arrival at the laboratory, homogenized soils were placed into pots, with each pot containing 700 g of soil. Two treatments (prophage induction and control) were included in this experiment. To ensure sufficient material for all analyses, six replicate pots were destructively sampled at each of the five timepoints, totaling 30 pots per treatment and 60 pots in total (Fig.  1). The soils were moistened to 60% water holding capacity, which was maintained throughout the incubation. For adjusting the initial soil moisture, mitomycin C solution was applied at the start of the experiment (time zero) to half of the pots at a final concentration of 0.5 μg·g^−1^ dry soil (labeled as the prophage induction treatment; PrIn). The remaining pots were irrigated with the same volume of sterile deionized water (serving as the control treatment; Ctrl). Each pot received the solution evenly to achieve uniform soil moisture content across treatments. Mitomycin C was applied only once at the beginning of the experiment, and no additional applications were made thereafter. The concentration of 0.5 μg·g^−1^ soil was selected based on previous studies demonstrating its efficacy in inducing prophage activation with minimal cytotoxic effects [7, 16]. All subsequent methodological details are provided below and in Supplementary materials (Section S1). Greenhouse-gas measurements were performed in parallel microcosms under matched treatments (see CO₂ and N₂O emission measurements).

Soybean seeds (*Glycine max cv.* Williams 82) were placed into the soil at a depth of approximately 2 cm in each pot. All pots were cultivated in an identical light box fitted with a neutral density gel filter to control light exposure at room temperature. The light treatment for each plant consisted of 16 h of light exposure at 100 lux and 8 h of darkness over a 35-day period in the chamber. The pots were destructively sampled on Days 7, 14, 21, 28, and 35, corresponding to the cotyledon (VC), first node (V1), second node (V2), third node (V3), and fourth node (V4) stages of soybean vegetative growth. These developmental stages represent key transitions in root and rhizosphere formation, which are critical for studying microbial colonization and nutrient interactions. At each time point, rhizosphere soil, bulk soil, roots, and shoot samples (including stems and leaves) in the pots were collected for downstream analyses. Specifically, six pot replicates (each containing three seedlings) of each treatment were destructively sampled at each sampling time. In total, 60 soil samples were collected during the pot experiment. The soil properties and bacterial community structure (via 16S rRNA gene amplicon sequencing) were analyzed in all collected soil samples. The virome community structure was determined in four soil samples (rhizosphere and bulk soil, under both prophage induction and control conditions) on the 28th day of the experiment (Fig.  1). To obtain sufficient viral DNA for virome sequencing, soil from three biological replicates per treatment at 28 days were pooled. This yielded four composite virome samples: BCtrl (bulk soil of control), RCtrl (rhizosphere of control), BPrIn (bulk soil of prophage induction), and RPrIn (rhizosphere of prophage induction).

### Soil properties and soybean root development analysis

The dissolved organic carbon (DOC), ammonium (NH_4_-N), and nitrate (NO_3_-N) contents in soil samples were immediately determined after sampling as previously described [17, 18]. DOC was measured to assess labile carbon pools and the carbon availability to microbes. NH_4_-N and NO_3_-N concentrations were determined to evaluate nitrogen mineralization and nitrification rates, which influence short-term nitrogen availability. Briefly, the NH_4_-N and NO_3_-N of each soil sample were extracted using 2 mol·L^−1^ KCL solution (with 5 g of soil suspended in 25 mL of solution), while the DOC in soil samples was extracted with deionized water at a soil-to-solution ratio of 1:5. The DOC concentration in the above extractions was measured on a total organic carbon and total nitrogen (TN) analyzer (MultiN/C 3000, Analytik Jena, Germany). To assess the impact of prophage induction on root development, the total length, average thickness, surface area, and volume of each soybean root were measured using Epson Expression 10000XL root system scanner (Seiko Epson) and analyzed with WinRHIZO Pro software.

A portion of each soil sample was air dried and ground before being sent out for determination of the content of the soil organic carbon (SOC), TN, and amino sugars in soil. SOC reflects the total carbon content in soil organic matter and indicates long-term carbon stability and carbon sequestration potential. TN represents the overall nitrogen reservoir and long-term nutrient supply capacity, encompassing both organic and inorganic forms. The SOC and TN values were used to calculate soil carbon–nitrogen stoichiometry (e.g., C/N ratio), which provides insight into soil nutrient balance and organic matter turnover. For analysis of the SOC and TN content in each sample, 300 mg of air-dried soil was analyzed using a Flash Smart Elemental Analyzer (Thermo Fisher Scientific). Amino sugars, essential structural components of microbial cell walls, are widely used as indices for the contribution of soil microbial necromass to SOC. Amino acids are released into the soil environment during decomposition of microbial residues and can be stably maintained in soil. Among all the identified amino acids, muramic acid (MurA), glucosamine (GluN), and galactosamine have been most often quantified in soil. MurA is exclusively present in bacterial cells, while fungal cell walls are major source of GluN in soil. Therefore, the calculation of bacterial and fungal residue C based on the composition of amino acids was proposed [19]. In brief, we determined microbial necromass as previously described by Zhang *et al*. [19]. The contents of amino sugars were then used to calculate bacterial and fungal necromass C contents according to the following equations [19]. Bacterial necromass C = MurA × 45; fungal necromass C = (GluN/179.17–2 × MurA/251.23) × 179.17 × 9. Total microbial necromass C = bacterial necromass C + fungal necromass C.

### Direct effects of mitomycin C on bacteria and plant root development

To evaluate the effects of mitomycin C on bacterial performance, we performed induction assays using three bacterial strains previously sequenced in our laboratory, i.e. *Stutzerimonas stutzeri* H46 (non-lysogen), *Chitinophaga* sp. EN-1 (non-lysogen), *Arthrobacter* sp. OA17 (lysogen), *Rhodococcus* sp. 1A6(lysogen), *Bacillus cereus* TJ-25 (lysogen), *Priestia megaterium* R-6 (lysogen), *Pseudomonas aeruginosa* FYS20 (lysogen), and *Klebsiella* sp. FX20–3 (lysogen). A laboratory reference strain, *Escherichia coli* DH5α, was also included as a non-lysogenic negative control in prophage induction assays. Detailed procedures for strain selection, prophage prediction, and mitomycin C induction assays are provided in Supplementary materials (Section S1). Each strain was incubated in 1× phosphate-buffered saline (PBS) supplemented with 0, 0.5, or 1 μg·mL^−1^ mitomycin C for 18 h, following the protocol described by [20]. After incubation, bacterial cells were enumerated using epifluorescence microscopy [20, 21], and the mortality rate of each strain was calculated based on cell counts.

The effects of mitomycin C on complex environmental microbial consortia were also investigated. Briefly, we collected soil and water samples and cultured them in minimal medium supplemented with 0, 0.5, or 1 μg·mL^−1^ mitomycin C. Bacterial growth dynamics were monitored by measuring OD_595_ growth curves, which served to assess both bacterial viability and potential prophage induction within the consortia.

To determine whether mitomycin C exerts a direct effect on plant growth, we conducted a parallel axenic experiment using *Arabidopsis thaliana* (ecotype Columbia-0) under sterile conditions. Seeds were surface-sterilized and germinated on Murashige and Skoog agar medium containing 0, 0.5, or 1 μg·mL^−1^ mitomycin C. Root length was measured at six time points (Days 4, 9, 11, 13, 16, and 22) to monitor plant developmental responses.

### CO_2_ and N_2_O emission measurements

Greenhouse gases, CO₂ and N₂O, emissions were quantified in parallel soil microcosms established with the same soil and treatment regime as the main pot experiment (control vs. prophage-induction; *n* = 3 per treatment). For each microcosm, 300 g dry-soil equivalent was placed into a 500 mL borosilicate bottle fitted with a butyl-rubber septum and three-way valve. Soils were adjusted to the same initial moisture as the pots and incubated at 25°C in the dark. Bottles were loosely capped for a 24 h pre-equilibration, then sealed during headspace sampling. Headspace gas (35 mL) was collected from each microcosm on Days 2, 4, 6, 8, 10, and 25 using gas-tight syringes, after gentle mixing (5 s) to homogenize the headspace. Samples were immediately transferred to pre-evacuated glass vials. Concentrations of CO₂ and N₂O were simultaneously analyzed using an Agilent 7890A gas chromatograph (Wilmington, DE, USA) equipped with a flame ionization detector and an electron capture detector. External multi-point standard curves (certified calibration gases spanning the sample range) were analyzed at the beginning and end of each run; linearity was *R*^2^ > 0.99. Instrument blanks and vial blanks were included every 10 samples.

### Virus quantification and virome sequencing

For virome sequencing, soil samples collected at Day 28 were selected from the rhizosphere and bulk soil compartments of both the prophage induction and control treatments. To ensure sufficient viral yield and DNA concentration, we pooled three biological replicates per group and used ≥15 g of composite soil for virus-like particles (VLPs) extraction. The 28-day timepoint for virome sequencing was selected because it represents a biologically informative stage where soybean roots are well-established, microbial and viral communities are stabilized, and root exudation is active. This timing also allows the cumulative effects of prophage induction to manifest in the virome, thereby enhancing the interpretability of treatment-driven viral community shifts. For extraction of VLPs from the soil matrix, the citrate–phosphate extraction buffer, composed of 1.44 g·L^−1^ Na_2_HPO_4_·7H_2_O, 0.24 g·L^−1^ KH_2_PO_4_, and 10 g·L^−1^ potassium citrate (pH at 7) was used as previously described [22]. The method for quantifying the extracted viruses was also elaborated in the above reference. Turbo deoxyribonuclease (DNase) I enzyme (Invitrogen) was used to remove unencapsulated DNA from the virus extractions. The VLPs in each sample were ultimately trapped on a 0.02-μm-pore-size Whatman Anodisc filter and were stained with SYBR gold nucleic acid dye at a final 2X concentration (Invitrogen, Carlsbad, CA, USA). The VLPs on the Anodisc filters were observed using an Olympus BX53 epifluorescence microscope with a FITC filter set (excitation at 467–498 nm and emission at 513–556 nm) and analyzed using Olympus cellSens imaging software.

Before virome sequencing, VLPs were extracted from each soil sample as described above, and the virus extractions were purified and concentrated by ultracentrifugation at 180 000 × g for 3 h at 4°C. The precipitated VLPs were resuspended in 400 μL sterile PBS and treated with 8 U of Turbo DNase I (Invitrogen) and 20 U of RNase A enzymes (Fermentas) at 37°C for 30 min to remove environmental DNA and RNA contamination. Viral nucleic acids were then extracted using QIAamp MinElute Virus Spin Kit (Qiagen) according to the manufacturer’s instructions [23]. The concentration and quality of the viral DNA samples were analyzed using NanoDrop2000c spectrophotometer (Thermo Scientific). DNA samples that met the quality criteria (OD260/280 = 1.8 ~ 2.2, OD260/230 ≥ 2.0) were sent to Shanghai Biozeron Biotechnology Co., Ltd. (Shanghai, China) for virome sequencing. The viromic library for each sample was prepared using a TruSeq Nano DNA sample preparation Kit (Illumina, San Diego, CA, USA). Metagenomic sequencing was performed on an Illumina NovaSeq 6000 in 150 bp paired-end (PE150) mode. The raw virome sequences for all samples were deposited in the Sequence Read Archive (SRA) at the National Center for Biotechnology Information (NCBI) under the accession number PRJNA1187349.

### Virome assembly and annotation

For virome analysis, we first used fastp v0.23.4 [24] to remove low-quality reads and adapter sequences. The PhiX sequences, a control library, were also removed during this process. High-quality reads were then assembled on a per-sample basis using three different software tools: MetaSPAdes v3.15.5 [25], MetaviralSPAdes v3.15.5 [26], and Penguin v5.cf8933 [27]. Assemblies were assessed using MetaQUAST v5.2.0 [28]. Phage contigs were predicted using two different tools: geNomad v20230721 [29] and Jaeger v1.1.23 (https://github.com/Yasas1994/Jaeger). These two tools were applied to the two contig sets generated by MetaSPAdes and Penguin, as MetaviralSPAdes already includes phage contig prediction. Thus, five sets of predicted viral contigs were generated for each sample (accession number of PRJNA1187349). These were then merged, combining all 20 contig sets from the four samples into a single file. The merged contigs were clustered into 52 374 vOTUs using vCLUST v1.0.0 [30], with a 95% ANI threshold and default single linkage. From each vOTU, we selected the best representative contig, and its quality was assessed using CheckV v1.0.1 [31] with the “end-to-end” command. To obtain auxiliary metabolic genes (AMGs), we followed the Viral sequence identification SOP with VirSorter2 V.3 from the Sullivan’ lab (dx.doi.org/10.17504/protocols.io.bwm5pc86), with few modifications. Briefly, we combined proviral and viral sequences predicted by CheckV into one file and used them as input to Virsorter2, starting from the “Run VirSorter2 again” step and skipping the initial run, as the viral sequences had already been predicted. The resulting tables and viral sequences were then used as input in DRAM-v [32]. For host prediction and additional functional annotation, we focused on 783 representative contigs with over 25% completeness and 0% contamination, as determined by CheckV. These were further annotated using iPHoP v1.3.3 [33] and Pharokka v1.7.1 [34].

### Read mapping and viral community composition

To determine the phage community composition, the metagenomic reads from the four samples were mapped against the complete combined set of metagenomic contigs using Bowtie2 v2.5.1 [35], and their coverage quantified using CoverM v0.5.0 (unpublished; https://github.com/wwood/CoverM). Reads were retained based on criteria of ≥95% identity and ≥ 75% read coverage. Counts of reads mapped to contigs were summed per cluster and normalized by sequencing depth of each sample. These normalized counts per cluster were used to estimate relative abundances by mapping clusters to the different functional and taxonomic annotations (i.e. AMG, IPHoP, Genomad).

### Soil bacterial DNA extraction, 16S rRNA gene sequencing and analysis

Soil bacterial DNA was extracted from the rhizosphere and bulk soils using the DNeasy PowerSoil Pro Kit (Qiagen, Hilden, Germany). The bacterial DNA extractions were quantified using a Nanodrop microvolume UV–Vis spectrophotometer (Thermo Scientific) and sent to Shanghai Lingen Biotechnology Co., Ltd. (Shanghai, China) for next-generation sequencing service. The V3–V4 region of bacterial 16S rRNA genes was amplified using barcoded primers (338F: 5'-ACTCCTACGGGAGGCAGCAG-3′; 806R: 5'-GGACTACHVGGGTWTCTAAT-3′). PCR products from each sample were quantified, purified, pooled, and sequenced using the Illumina MiSeq platform with 300 bp paired-end reads.

The sequence files obtained were demultiplexed and sorted into corresponding samples based on unique barcodes. The raw sequence data for each sample was processed using the QIIME2 pipeline (version 1.16) [36]. In general, the quality profiles of all reads were assessed using DADA2, which removed low-quality and chimeric sequences. The remaining sequences were clustered into OTUs, and taxonomic annotation (at domain-, phylum-, class-, order-, family-, genus-, and species-level) was assigned to each representative OTU using the naive Bayesian classifier method based on the classify-sklearn algorithm in QIIME2. All statistical analyses and visualization were performed using R version 4.3.1 [37]. Microbial ecological analysis was conducted using the “microeco” package [38]. The raw bacterial 16S rRNA gene sequences for all samples were uploaded to the SRA at NCBI (accession number of PRJNA1148935).

### Minimal doubling time predictions

We estimated bacterial growth rate potential using the Estimated Growth rates from gRodon Online (EGGO) database [39] based on the predicted minimum doubling times (PMDTs). EGGO database contains growth rate predictions for over 217 000 prokaryotic genomes (including metagenome-assembled genomes and single-amplified genomes) using codon usage models. For each identified genus, we computed the median PMDT (mPMDT) based on the affiliated genomes in the EGGO database.

### Statistical analysis

The Chao1 and Shannon indices were calculated to estimate bacterial species richness and community diversity, respectively. Principal co-ordinates analysis (PCoA) was performed to calculate the dissimilarities of bacterial communities from rhizosphere and bulk soil samples of different treatment based on Bray-Curtis distance, permutational multivariate analysis of variance (PerMANOVA) was used to analyse statistical differences between treatments in the “vegan” package [40]. Differentially abundant taxa were first evaluated using the Kruskal-Wallis (KW) H test. To improve statistical robustness and account for the compositional and sparse nature of microbiome data, we further applied Multivariable Association with Linear Models (MaAsLin2), in the “maaslin2” package) for multivariate differential abundance analysis. Input data were normalized using total sum scaling, and the model included treatment and compartment as fixed effects. Significant associations were identified using FDR-corrected *q*-values <0.05 [41]. The correlation of soil physicochemical properties with bacterial community structure was analyzed using distance-based redundancy analysis (dbRDA) based on Mantel’s test and Pearson correlation in the “microeco” package. Linear discriminant analysis Effect Size (LEfSe) used the non-parametric factorial KW sum-rank test, unpaired Wilcoxon rank-sum test and linear discriminant analysis (LDA) to estimate the effect size of each differentially abundant feature [42]. The bacterial interaction network was calculated by Molecular ecological network analysis through Sparse Correlations for Compositional data method [43]. The keystone species in the bacterial interaction network were determined by within-module connectivity (*z_i_* > 2.5) and among-module connectivity (*P_I_* > .625), all the methods and statistical tools are openly-accessible in (https://inap.denglab.org.cn/) and visualization of results in the software Gephi [44]. The means of the data were used to represent the result in each treatment at a specific sampling time point, with standard deviations calculated. *Post hoc* test (Duncan’s multiple range test) with analysis of variance (ANOVA) was used to calculate statistical differences between treatments. KW and student’s *t*-test were used to calculate statistical differences between two treatments in Statistical Product and Service Solutions (SPSS). The images were visualized using R package “ggplot2” [45] and Origin 2021.

## Results

### Prophage induction alters viral abundance, virome composition, and function

**Figure 2:**
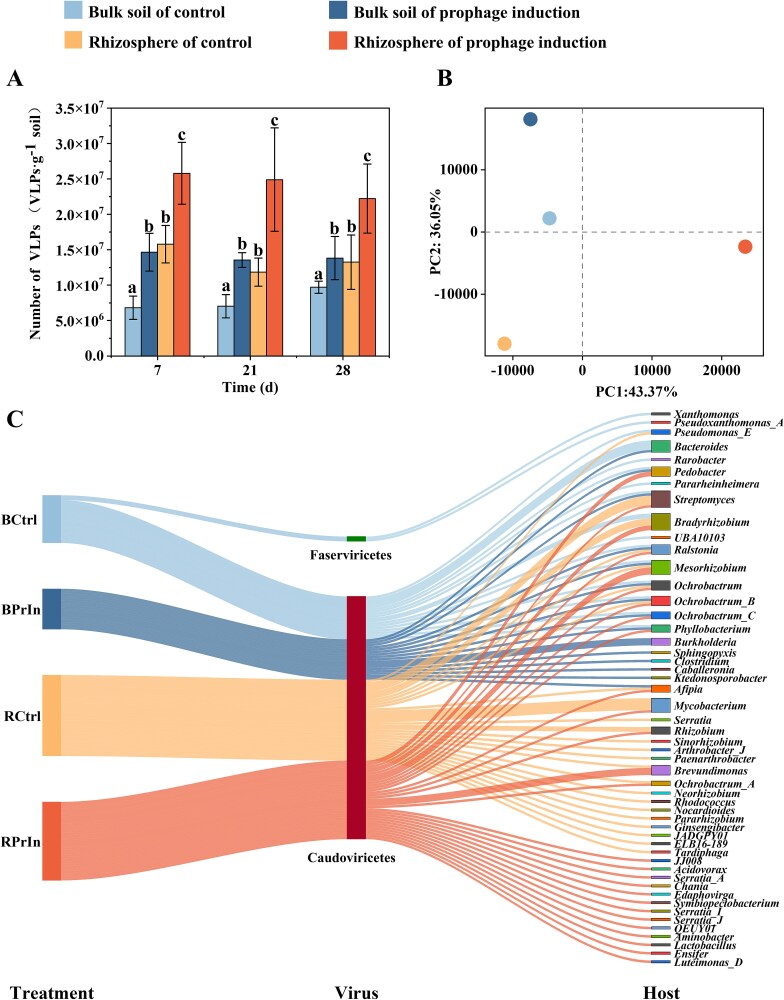
The viral community dynamics in bulk soil and rhizosphere from both control and mitomycin C induction treatment. (A) The VLP abundance as influenced by prophage induction. The data show the mean value of triplicate samples, error bars show standard deviation. Statistical differences between groups were determined according to *post hoc* test (Duncan’s multiple range test) with ANOVA. The statistical significance was indicated by letters, a, b, and c (*P* < .05). (B) Principal components analysis of viromes, each point represents one sample on Day 28. (C) The relationship between viruses and their corresponding hosts from different treatments. There are 783 representative contigs with over 25% completeness and 0% contamination for host prediction. The middle and right side shows the virus at class level and their associated host at genus level. The lines connect the virus to host bacteria, the color of the lines represents different treatments, and the width of the lines represents the number of contigs of the virus and host bacteria. Treatments are: BCtrl, Bulk soil of control; BPrIn, Bulk soil of prophage induction treatment; RCtrl, Rhizosphere of control; RPrIn, Rhizosphere of prophage induction treatment.

Mitomycin C is widely used as a prophage inducing agent for studying lysogenic populations. Low concentrations of mitomycin C (0.5 ~ 1 μg per mL culture) have negligible toxic effects on microbial cells but can effectively induce lysogeny-to-lysis conversions [6, 7]. Strain-level responses to mitomycin C were further evaluated in eight soil bacterial isolates, including both lysogenic and non-lysogenic representatives (see Supplementary materials, Section S1 for strain selection and induction protocols). Our experiments demonstrated that mitomycin C treatment resulted in a mortality rate of less than 23% in the non-lysogenic strain *S. stutzeri* H46 (21%), *Chitinophaga* sp. EN-1 (22%), and *E. coli* DH5α (21%), whereas significantly higher mortality rates were observed in the other six lysogenic strains (Arthrobacter sp. OA17: 98%; *Rhodococcus* sp. 1A6: 44%; *B. cereus* TJ-25: 67%; *Priestia megaterium* R-6: 99%; *P. aeruginosa* FYS20: 93%; *Klebsiella* sp. FX20–3: 34%) under treatment with 0.5 μg·mL^−1^ mitomycin C (Supplementary Fig. S1A). In contrast, the growth performance of environmental microbial consortia derived from soil samples was not significantly inhibited by 0.5 μg·mL^−1^ mitomycin C (Supplementary Fig. S1B), indicating minimal direct toxic effects of the treatment on environmental bacterial cells. The VLP abundance in the bulk soil of the control treatment increased throughout the cultivation period (ranging from 6.8 × 10^6^ to 9.7 × 10^6^ VLPs per g of soil, *P* = .085, ANOVA, Fig.  2A). Soil samples following mitomycin C induction treatment contained significantly higher VLP abundance (1.3 × 10^7^ to 1.9 × 10^7^ VLPs per g of soil) than those of the control (*P* < .01), suggesting that prophage induction was effective. The rhizosphere sustained higher VLP abundance (1.2 × 10^7^ to 2.6 × 10^7^ VLPs per g of soil) than the bulk soil, further increased by the mitomycin C induction treatment (Fig.  2A). Chemical induction thus caused intensified viral-mediated cell lysis and VLP production in both bulk soil and rhizosphere.

To gain further insight into the viral populations across treatments, we obtained virome-enriched metagenomic samples of rhizosphere and bulk soils at 28 days, sequenced them, and analyzed their functional and taxonomic content. We obtained approximately the same number of high-quality virome enriched metagenomic reads across all samples (Supplementary Table S1). A total of 199 439 putative viral contigs were obtained using three different assemblers (see Methods). CheckV results showed most of these predicted viral contigs were short (<10 000 bp) and incomplete (<25%), as expected for diverse and complex soil samples (Supplementary Fig. S2A). About 21 712 representative contigs were taxonomically annotated up to the class level. Read mapping revealed that Caudoviricetes, by far the most abundant class, slightly decreased in relative abundance upon prophage induction (Supplementary Fig. S2B). The species richness and diversity of the viromes decreased from bulk soil to rhizosphere in the control. Compared with the control, mitomycin C induction increased viral species richness and diversity in the rhizosphere but decreased it in the bulk soil (Supplementary Table S2). Principal component analysis suggested that the induction treatment promoted the differentiation of the rhizosphere virome from bulk soil, and rhizosphere viromes were more shifted than those in bulk soils (Fig.  2B).

We then detected AMGs in viral contigs (see Methods), which resulted in 328 predicted AMGs present in 137 different vOTUs (Supplementary Data S1 and Fig. S2C). When we evaluated the reads that mapped to these functional modules, we found glycosyltransferases, methionine degradation, and pyrimidine deoxyribonuleotide biosynthesis to be highly abundant in rhizosphere viruses. In the rhizosphere, glycosyltransferases, specifically GT2 cellulose synthase, which was the only detected gene in this module, may be important for the establishment of bacterial biofilms and the interaction with plants [46]. Methionine degradation may be important as a carbon and nitrogen source that could process methionine release in plant exudates [47]. Finally, the module pyrimidine deoxyribonuleotide biosynthesis included the enzymes dUTP pyrophosphatase, ribonucleoside-diphosphate reductase, dCTP deaminase, and thymidylate synthase, which play crucial roles in nucleotide metabolism and are essential for bacterial growth and proliferation, particularly in nutrient-rich environments like the rhizosphere [48]. Interestingly, we found the module Glycoside Hydrolases more abundant in the RPrIn treatment, suggesting the utilization of complex sugars is a trait that may be enriched upon prophage induction.

Functional annotations in all representative contigs from the four samples resulted in approximately 52.5–87.2 thousand CDS, with the vast majority (86.3–87.5%) being annotated as unknown function (Supplementary Table S3 and Fig. S2E). From the top 20 more abundant functions within the category “moron, auxiliary metabolic genes, and host take-over”, we observed “glycosyltransferase”, “ribosomal protein S6 glutaminyl transferase”, “cyanobacterial phosphoribosylglycinamide formyltransferase” being more abundant in the rhizosphere induced viromes (Supplementary Fig. S2F).

We then selected those representative contigs with more than 25% estimated completeness, obtaining 183, 145, 186, and 269 contigs for BCtrl, BPrIn, RCtrl, and RPrIn, respectively, representing 0.3% of the virome reads. We predicted putative hosts for these viral contigs (Fig.  2C) and evaluated their growth rate by estimating mPMDT (see Methods). We could not observe any preference for fast or slow growing bacteria in this subset of the viromes (Supplementary Fig. S2D).

### Enhanced root development in response to prophage-induced nutrient changes

**Figure 3:**
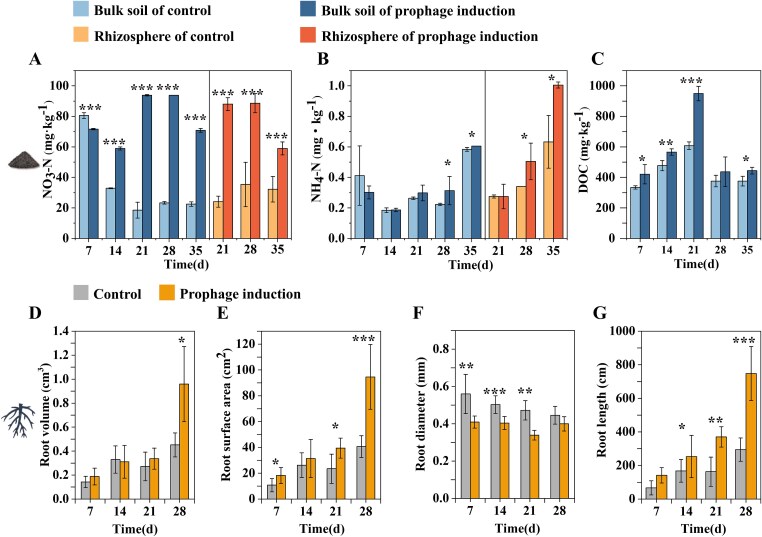
The soil nutrient conditions and root development. The content of NO_3_-N (A), NH_4_-N (B), and DOC (C) in the rhizosphere and bulk soils were examined. The root volume (D), surface area (E), diameter (F), and length (G) were measured. The data of soil properties show the mean value of triplicate samples, and the data of root development results represent the mean value of quintuplicate samples. Error bars show standard deviation. Statistical differences between different treatments were determined according to Student’s *t*-test, and the statistical significance is labeled accordingly (^*^P < .05; ^**^P < .01; ^***^P < .001).

Prophage induction caused significant changes in nutrient levels in both the rhizosphere and bulk soil. In the control treatment, nitrate content ranged from 18.5 to 80.5 mg·kg^−1^ in bulk soil and from 24.1 to 35.3 mg·kg^−1^ in the rhizosphere (Fig.  3A). Following mitomycin C treatment, the nitrate content ranged from 58.9 to 93.8 mg·kg^−1^ in bulk soil and from 58.9 to 88.6 mg·kg^−1^ in the rhizosphere. Prophage induction initially decreased nitrate content in bulk soil at 7 days but then increased nitrate content from 14 days to the end of the experiment (*P* < .001). Additionally, prophage induction increased NH_4_-N content in the rhizosphere (*P* < .05 at 28 days and 35 days) and in bulk soil (*P* < .05 at 35 days; Fig.  3B). The soil DOC content was notably higher upon induction (ranging from 421.2 to 949.6 mg·kg^−1^) than in the control samples (ranging from 334.3 to 607.7 mg·kg^−1^) after three weeks (*P* < .001; Fig.  3C), but this effect diminished after four weeks. Prophage induction had little effect on soil TN and SOC content, while the carbon/nitrogen ratio was slightly decreased (Supplementary Fig. S3A−C).

Specifically, compared to the control treatment, the prophage induction treatment increased root volume, length, and surface area by approximately two-fold throughout the experiment, accompanied by a slight reduction in average root diameter (Fig.  3D−G). This suggests that the plant responds to the changes in carbon and soil content by expanding its root system. The direct effects of mitomycin C on plant root development were assessed in a parallel axenic experiment using *A. thaliana* (Columbia-0 ecotype). Across all timepoints and concentrations, there were no significant differences in root length or morphology among treatments (Supplementary Fig. S4), indicating that mitomycin C, at concentrations comparable to those used in the soil experiment, did not impair root growth or cause observable phytotoxicity under sterile conditions. This supports the interpretation that the root trait changes observed in the soybean experiment are likely mediated indirectly through microbial interactions rather than a direct effect of mitomycin C on plant physiology (see Supplementary materials, Section S2).

### Phage-induced shifts in bacterial community structure over time

**Figure 4:**
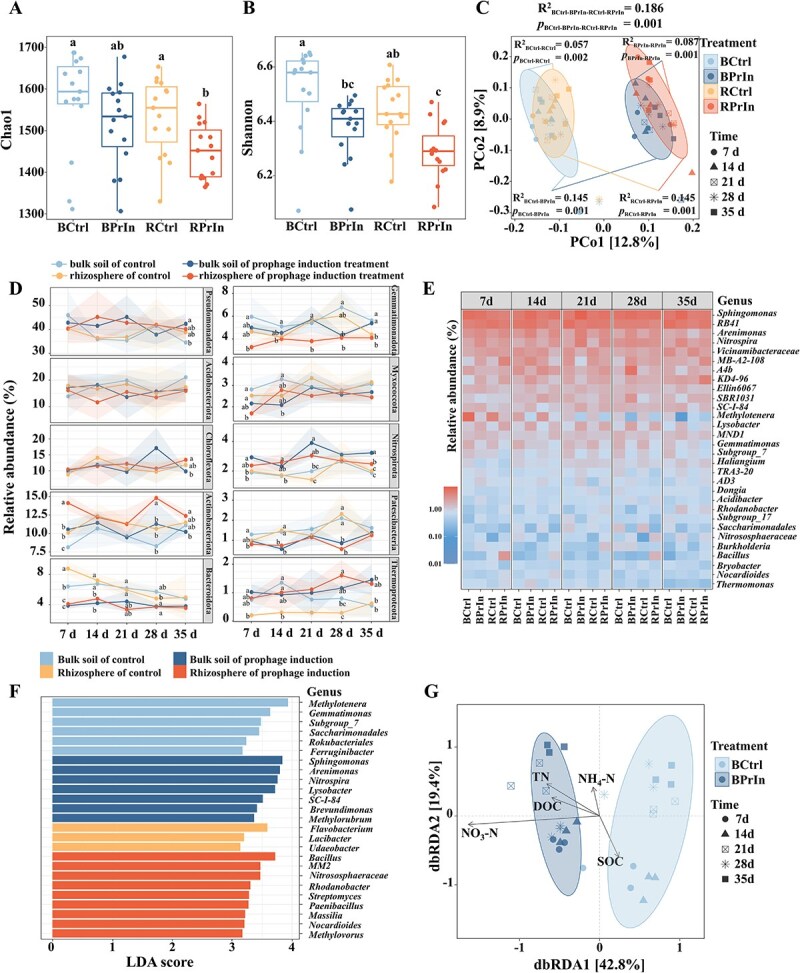
The impact of prophage induction on the bacterial community properties. (A) Chao1 richness index of bacterial communities in the rhizosphere and bulk soils. (B) Shannon diversity index of bacterial communities in the rhizosphere and bulk soils. There are 15 samples in each treatment, and statistical differences between treatments were determined according to *post hoc* test (Duncan’s multiple range test) with ANOVA. The statistical significance was indicated by letters, a, b, and c (*P* < .05). (C) PCoA of bacterial community structure dissimilarities based on bray-Curtis distances. PerMANOVA was used to analyse statistical differences between treatments, and each point represents a sample with the color indicating treatment, the shape indicating sampling time, and the elliptical circles indicating the confidence ellipse of each treatment at 90% confidence level. (D) The taxonomic composition of bacterial communities at phylum level during incubation. Each point represents the mean value of triplicate samples, the shaded area depicts the mean ± standard deviation over the triplicate samples. Statistical differences between groups were determined according to *post hoc* test (Duncan’s multiple range test) with analysis of variance (ANOVA). The statistical significance was indicated by letters, a, b, and c (*P* < .05). (E) Heatmap of the top 30 bacterial genera in rhizosphere and bulk soils. (F) LDA effect size analysis (LEfSe analysis) of top 25 bacterial genera enriched in the rhizosphere and bulk soils or under prophage induction. (G) dbRDA of the effects of soil nutrient conditions on bacterial community structure in bulk soil. Each point represents a sample with the color indicating treatment and the shape indicating sampling time. The elliptical circles indicate the confidence ellipse of each treatment at 90% confidence level, and the length of the line connecting the arrow to the origin represent the magnitude of the correlation between the explanatory matrix and bacterial community structure. Treatments are: BCtrl, Bulk soil of control; BPrIn, Bulk soil of prophage induction treatment; RCtrl, Rhizosphere of control; RPrIn, Rhizosphere of prophage induction treatment.

In general, the bacterial species richness (indicated by Chao1) and community diversity (indicated by Shannon) in the rhizosphere were lower than in the bulk soil, especially following prophage induction (*P* < .05, Duncan’s test; Fig.  4A and B). Prophage induction, accompanied by high viral production and abundance, and shifted virome structure, significantly reduced bacterial community diversity and species richness in both bulk soil and rhizosphere (Supplementary Fig. S5A−D), exerting substantial impacts on the bacterial community structure. In the rhizosphere, prophage induction consistently decreased bacterial species richness and community diversity throughout the experiment (*P* < .05 at Days of 7, 28, and 35; Supplementary Fig. S5C and D). The bacterial assemblages from rhizosphere and bulk soils under prophage induction were analyzed with PCoA (Fig.  4C). The bacterial communities in the control treatment were strongly separated from those in the mitomycin C-induction treatment (based on PerMANOVA, Fig.  4C), suggesting prophage induction was a significant factor in shaping the bacterial communities in both bulk soils (*R*^2^ = 0.15, *P* = .001; Fig.  4C) and rhizosphere (*R*^2^ = 0.15, *P* = .001; Fig.  4C). Differentiation of rhizosphere bacterial communities from those in bulk soil was observed both in the control (*R*^2^ = 0.06, *P* = .002, PerMANOVA; Fig.  4C) and induction treatment (*R*^2^ = 0.09, *P* = .001; Fig.  4C).

dbRDA, which addresses the effect of an explanatory matrix (e.g., environmental variables) on a response matrix (i.e., the bacterial community) using Euclidean distances, was performed to assess the effects of soil nutrient conditions on the bacterial community assembly process in bulk soil. The NH_4_-N, NO_3_-N, and SOC content significantly influenced bacterial community structure under prophage induction, with NO_3_-N content exerting the strongest effect (*r* = 0.4, *P* < .01; Mantel test; Fig.  4G).

In total, 37 bacterial phyla were detected in the experimental system, among which Pseudomonadota (relative abundance ranging from 35.4% to 44.9%), Acidobacteriota (11.3–21.1%), Actinobacteriota (8.1–14.6%), Chloroflexota (9.5–17.1%), Bacteroidota (3.4–8.7%), Gemmatimonadota (3.2–6.8%), Nitrospirota (1.4–3.9%), and Myxococcota (1.6–3.3%) were the most abundant bacterial phyla across all the soil samples (Fig.  4D). The taxonomic composition of the bacterial communities in the rhizosphere and bulk soil was significantly affected by prophage induction, and significant differences in relative abundances of bacterial taxa between different samples were identified based on statistical analysis using the ANOVA (cutoff of *P* < .05). The relative abundance of Acidobacteriota, Bacteroidota, Gemmatimonadota, Methylomirabilota, Patescibacteria, and Verrucomicrobiota in the rhizosphere decreased following induction treatment, while the relative abundance of Nitrospirota, Bacillota, Thermoproteota, and Pseudomonadota increased (Fig.  4D). Detailed relative abundances and corresponding statistical significance values (*P* < .05) for these taxa are provided in Supplementary Data S2. Differential abundance analysis using MaAsLin2 identified treatment-specific enrichment patterns consistent with KW results (Supplementary Table S4). Notably, Actinobacteriota was significantly enriched in the prophage-induced rhizosphere communities (*P* < .05). The impact of prophage induction on bacterial community composition at class level was analyzed in Supplementary Fig. S6.

At finer taxonomic resolution, genus-level differences are shown in Fig.  4E, which highlights the 35 most abundant genera across treatments. *Sphingomonas* was the most abundant genus in all treatments, and its relative abundance remained stable through the experiment and across all treatments (ANOVA, *P* > .05). Prophage induction increased the proportion of *Arenimonas*, *Nitrospira*, *Lysobacter*, *Rhodanobacter*, and *Thermomonas*, while decreasing that of *A4b*, *Methylotenera*, *Gemmatimonas*, *Subgroup_7*, *Saccharimonadales*, and *Bryobacter* in both rhizosphere and bulk soil (*P* < .05, ANOVA; Fig.  4E). The bacterial genera *SC-I-84*, *Nitrososphaeraceae*, *Burkholderia-Caballeronia-Paraburkholderia*, and *Bacillus* were 2- to 7-fold enriched by prophage induction in the rhizosphere. LEfSe analysis was performed to assess the differentiation of bacterial communities across different treatments. The bacterial communities in the rhizosphere following prophage induction were characterized by *Rhodanobacter*, *Streptomyces*, *Paenibacillus*, *Massilia*, *Nocardioides*, *Methylovorus*, and *Polycyclovorans*. In contrast, those in the bulk soil of the induction treatment were characterized by *Sphingomonas*, *Arenimonas*, *Nitrospira*, *Lysobacter*, *SC-I-84*, *Brevundimonas*, and *Methylotenera* (Fig.  4F).

**Figure 5:**
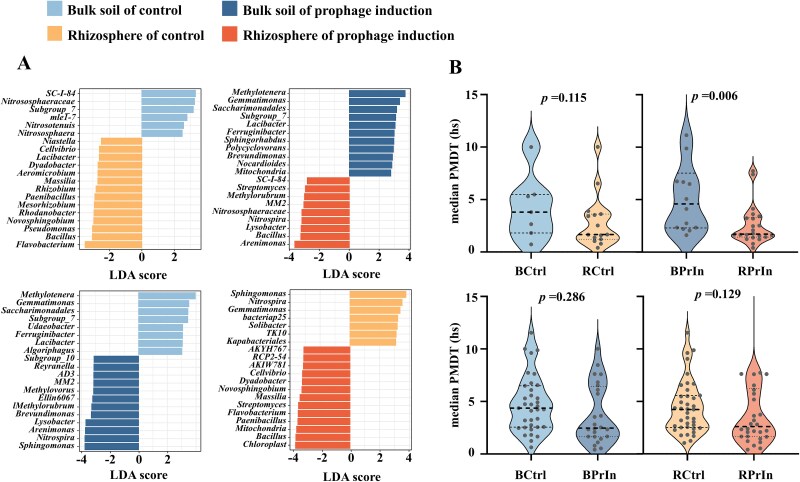
The impact of prophage induction on the bacterial median predicted minimal doubling times (mPMDT) (A) pairwise comparison LDA effect size analysis (LEfSe analysis) of top 20 bacterial genera for identification of enriched genera in the rhizosphere and following prophage induction. (B) Predicted minimal doubling time in enriched genera from LEfSe analyses. Comparisons were made between BCtrl-RCtrl, BPrIn-RPrIn, BCtrl-BPrIn, and RCtrl-RPrIn treatments, and significantly enriched genera (LEfSe, *P* < .05) were used to map median predicted minimal doubling times. Mann–Whitney test *P* values are shown for mPMDT comparisons. Treatments are: BCtrl, Bulk soil of control; BPrIn, Bulk soil of prophage induction treatment; RCtrl, Rhizosphere of control; RPrIn, Rhizosphere of prophage induction treatment.

Pairwise comparison LEfSe analysis determines the features most likely causing differences between groups with statistical significance [42]. We used this test to analyse the top 20 bacterial genera in different soils. The pairwise comparison analysis, i.e. BCtrl-BPrIn, BCtrl-RCtrl, BPrIn-RPrIn, and RCtrl-RPrIn, revealed enrichment of specific bacterial genera in rhizosphere and following mitomycin C induction (Fig.  5A). We then analyzed the enriched genera obtained from LEfSe comparisons between treatments (KW test, *P* < .05) and estimated their growth rate (see Methods) to evaluate whether viral induction disproportionally affected fast-growing bacteria, as observed in the rhizosphere effect [1, 49]. In the control treatment, we could not confirm the significant enrichment of fast-growing bacteria in the rhizosphere, nor did prophage induction lead to their relative enrichment. We did observe significant enrichment of fast-growers in the rhizosphere in the mitomycin C-treated experiments, suggesting that prophage induction catalyses a viral shunt that strengthens the plant exudate-driven nutrient gradient, favoring fast-growing bacteria and enhancing the rhizosphere effect (Mann–Whitney test *P* < .05 in BPrIn vs. RPrIn; Fig.  5B). The enrichment of fast-growing bacterial populations in rhizosphere was stimulated by prophage induction, as fast growers, such as the relative abundance of *Bacillus*, *Rhodanobacter*, *Streptomyces*, *Paenibacillus*, *Massilia*, *Nocardioides*, *Methylovorus*, and *Polycyclovorans*, were particularly increased by viral activities (Fig.  5B).

**Figure 6:**
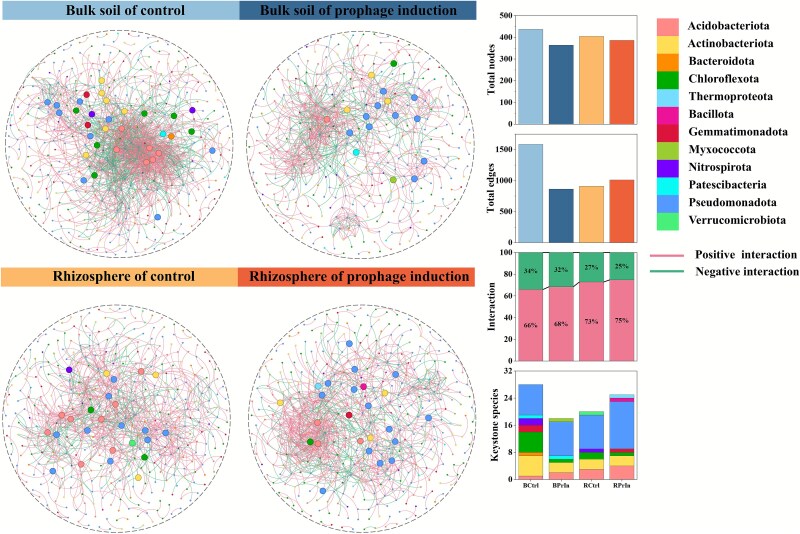
The co-abundance networks in the rhizosphere and bulk soils. The larger nodes indicate keystone species based on the within-module connectivity (*z*_i_ > 2.5) and among-module connectivity (*P*_i_ > .625). Green lines between nodes (edges) indicate positive interactions, and pink lines indicate negative interactions. Treatments are: BCtrl, Bulk soil of control; BPrIn, Bulk soil of prophage induction treatment; RCtrl, Rhizosphere of control; RPrIn, Rhizosphere of prophage induction treatment.

Elucidating the complex relationships within bacterial communities based on 16S rRNA gene sequencing data provides the possibility to better understand bacterial community assembly mechanisms in different treatments. In the untreated control, the complexity of the bacterial interaction network decreased in the rhizosphere (405 nodes, 910 edges −27.1% negative interactions) compared to the bulk soil (437 nodes, 1586 edges −34.2% negative interactions) (Supplementary Table S5 and Fig.  6), reflecting the differences in diversity. Prophage induction substantially shifted the interaction patterns, further differentiating the rhizosphere bacterial community from that of bulk soil. Mitomycin C induction decreased the complexity of the bulk soil network and reduced the proportion of negative edges from 34.2% to 31.6%. Conversely, the complexity of the rhizosphere network was increased under prophage induction, with 25.1% negative edges compared to 27.1% in the control (Supplementary Table S5 and Fig.  6).

### Prophage induction modulates carbon mineralization and microbial necromass accumulation

**Figure 7:**
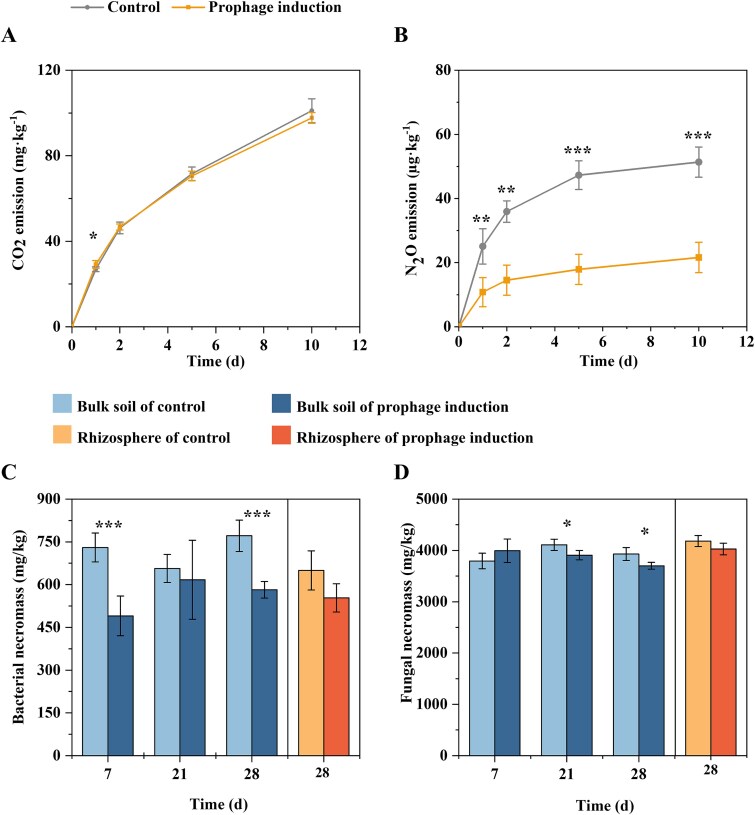
The impact of prophage induction on carbon and nitrogen mineralization and microbial necromass accrual. (A) The production of CO_2_ (A) and N_2_O (B) as influenced by prophage induction. A separate soil microcosm experiment was conducted to inspect the CO_2_ and N_2_O production in soil. The bacterial necromass (C) and fungal necromass (D) in the rhizosphere and bulk soils were calculated from the measured content of soil amino sugars. Statistical differences between different treatments were determined according to Student’s *t*-test, and the statistical significance is indicated by asterisk symbols, i.e. ^*^P < .05; ^**^P < .01; ^***^P < .001. Treatments are: BCtrl, Bulk soil of control; BPrIn, Bulk soil of prophage induction treatment; RCtrl, Rhizosphere of control; RPrIn, Rhizosphere of prophage induction treatment.

The effect of mitomycin C induction on organic carbon mineralization was examined by monitoring the greenhouse gas (i.e. CO_2_ and N_2_O) cumulative emissions during a separate soil incubation (Fig.  7A and B). Prophage induction did not affect CO_2_ production, but N_2_O emission was decreased by 2.6-fold (47.3 g·kg^−1^ for Control and 17.9 μg·kg^−1^ for the prophage induction treatment). At the late stage of incubation, no significant difference was observed in N_2_O production rate between control and prophage induction treatment.

Prophage induction directly contributes to bacterial mortality and likely increases bacterial necromass production, recycling, and carbon accumulation in soil. The content of amino sugars in the pot soils indicated that the bacterial and fungal necromass content in the mitomycin C-induced treatments was consistently less than that of the control in both bulk and rhizosphere samples (*P* < .01; Fig.  7C and D). Together, these results delineate a treatment-specific restructuring of rhizosphere functions that is consistent with increased viral production and altered nutrient turnover.

## Discussion

Understanding the mechanisms through which prophage induction alters microbial community dynamics and plant performance is critical for unraveling virus-mediated processes in soil ecosystems. Our investigation into prophage induction and its effects on rhizosphere microbial communities, nutrient cycling, and soybean root development reveals potential broader ecological significance. While the mechanisms by which prophage induction influences nutrient cycling and plant performance remain to be further elucidated, we note a parallel between the effects of mitomycin C on soybean root development and the use of antibiotics as growth promoters. A body of research of several decades supports the notion that antibiotics improve growth efficiency and yield, especially in the livestock industry. Proposed mechanisms include a reduction of nutrient competition with the host, reduced disease incidence by inhibition of pathogens, modification of the microbiome to a more beneficial profile, stimulation of the microbes by hormesis, and direct stimulation of host cells [50–52]. Microbe-free plant tissue culture and seed germination were also stimulated by low levels of antibiotics [51, 53]. To our knowledge, the release of nutrients by prophage induction, triggered by subinhibitory antibiotics concentrations has not yet been proposed as a potential mechanism. Our findings with the DNA crosslinking agent mitomycin C suggest that this may also increase nutrient availability in the rhizosphere and benefit the plant. While commonly used antibiotic growth promoters have different mechanisms of action, many can trigger prophage induction [54, 55]. Thus, we propose that the subsequent viral shunt may serve as a novel mechanism for antibiotics-stimulated growth promotion of higher hosts, including organisms of agricultural importance such as plants and livestock. Notably, future research should explore this hypothesis further, especially because application of antibiotics in agriculture has already contributed significantly to the global antibiotics resistance crisis [56] and is thus undesirable as we strive to reduce antibiotics use.

### Soil viromes linked to prophage induction

By examining the viral community dynamics after prophage induction with mitomycin C, we demonstrated that chemical induction led to a sharp increase in VLP abundance (Fig.  2A) and a potential increase in overall viral diversity in the rhizosphere (Supplementary Table S2), even though some viral lineages were not detected among the representative contigs shown in Fig.  2C. VLP abundances were increased by 1.4-fold to 2.1-fold in bulk soils and by 1.6-fold to 2.1-fold in the rhizosphere following mitomycin C induction (Fig.  2A). This significant increase in viral abundance suggests extensive lysogeny-to-lysis switching among prophages upon mitomycin C induction.

Viromes in bulk soils and rhizosphere were dominated by Caudoviricetes. Despite similar responses of viral community dynamics to mitomycin C induction in the rhizosphere and bulk soils, induction of prophages caused more variations in rhizosphere viral community than in bulk soil and increased the dissimilarities between rhizosphere and bulk soil viromes (Fig.  2B). The mitomycin C-induced differentiation of the rhizosphere virome from bulk soil is likely due to the production of new phages from previously lysogenized populations. The increased viral community diversity and species richness in the rhizosphere under prophage induction (Supplementary Table S2) demonstrated that a large fraction of viral populations exist in the form of prophages which may play a pivotal role in maintaining viral community diversity in the rhizosphere. Previous studies have shown that prophages are widespread among environmental bacteria. Prophage diversity and function are largely unknown [6, 57, 58]. For example, Roux *et al*. [43] identified 12 498 high-confidence phage genomes from 14 977 bacterial/archaeal genomes in the NCBI RefSeq database, augmenting the public viral genomic data sets by 10-fold. These results suggest that diverse prophages are widely present in bacterial/archaeal genomes and potentially play a critical role in driving microbial processes. Previous observations suggested that lysogeny is favored in the rhizosphere as a major portion of phages exist as prophages within rhizobacterial cells [14, 15], thus it is important to investigate the baseline prophage diversity and function in plant rhizosphere bacteria and to define whether or not transitions to lysogeny occur upon the selective filtering of the soil microbial community to the lower taxonomic diversity observed in the rhizosphere. Here, we defined a transition from lysogeny to lytic phage replication in soil and rhizosphere environments. We also demonstrated that the extensive lysogeny-to-lysis switch can cause a substantial increase in phage production, increasing rhizosphere phage species richness. These results demonstrate that prophages account for an important proportion of rhizosphere phage communities, which can be modulated in response to environmental cues.

### Rhizobacterial community dynamics and differentiation under viral predation

Recruitment of beneficial root-associated bacterial communities in the rhizosphere sustains plant growth and ecosystem productivity [10, 13]. Bacterial members of the rhizosphere are selected through niche filtering, creating a crucial microbial hotspot in the rhizosphere, which results in a distinct rhizobacterial community structure compared to that of bulk soil [59, 60]. Recently, bacterial growth rate potential was identified as the main trait for rhizosphere recruitment [61]. In our assays we observed a significant enrichment of the fast-growing bacteria in the rhizosphere under prophage induction (Fig.  5B). This observation may be related to the improved nutrient conditions (Fig.  3A−C), as the increased nutrients via “viral shunt” can particularly favor r-strategist bacteria, the fast growers [62]. The result is also in accordance with the “Piggyback-on-the-Winner” model, which suggests phages are prone to maintain lysogenic cycles under high nutrient conditions and microbial density by piggybacking in the genomes of the dominant bacterial populations [63]. The core rhizosphere bacteriome across various plant types includes Pseudomonadota, Bacteroidota, Bacillota, and Actinobacteriota, the four largest, generalist bacterial phyla [64], and Acidobacteriota. These groups are associated with nutrient cycling, organic matter decomposition, and plant growth stimulation [65]. Here, we demonstrated that prophage induction specifically increased the relative abundance of some major bacterial genera such as *Nitrospira*, *Lysobacter*, *Paenibacillus*, *Bacillus*, *Massilia*, *Sphingomonas*, *Arenimonas*, and *Methylorubrum* in the rhizosphere (Fig.  4E). Many of these genera contain plant-growth-promoting members [66, 67]. The treatment-specific changes observed in alpha diversity (Chao1 and Shannon indices; Supplementary Fig. S5) suggest that prophage induction may differentially impact microbial species richness and evenness depending on soil compartment. In the rhizosphere, where root exudation fuels microbial growth and interspecies interactions, prophage-mediated lysis may disproportionately target dominant or fast-growing taxa [16], disrupting community balance and leading to temporary declines in diversity. This effect may be enhanced by the higher frequency of lysogeny in nutrient-rich environments such as the rhizosphere [63]. In contrast, microbial communities in bulk soil, with lower nutrient availability and reduced microbial density, may be less responsive to prophage induction and maintain preserving baseline diversity levels. These findings are consistent with context-dependent phage–host dynamics and ecological models that highlight selective lineage suppression through phage activity [68].

The increased viral production that can cause more intense bacterial mortality and community turnover, enhanced soil nutrient conditions, leading to enrichment of dominant, fast-growing rhizosphere-enriched bacterial taxa (Fig.  3A−C and Fig.  5). The increased domination of copiotrophs can suppress low-abundance populations and facilitate the exclusion of species from the community, thereby reducing both the community diversity and species richness [69]. Phages and their microbial hosts are intimately intertwined and variations in viral community structure and activities will lead to notable changes in bacterial communities [70]. The importance of Pseudomonadota and Bacillota in the rhizobacterial interaction network was seemingly increased, while that of Nitrospirota was seemingly decreased following prophage induction (Fig.  6 and Supplementary Fig. S7). This suggests that increased viral predation within bacterial taxa that commonly undergo lysogeny-to-lysis transition can contribute to the enrichment of the major bacterial taxa in the rhizosphere. Previous studies demonstrated that both interspecific competition and cooperative interactions like metabolite exchanges have critical roles in bacterial community assembly [71, 72]. Viral lysis, capable of releasing physiologically relevant formerly intracellular nutrients into the environment, may transform the current inter-species relationships and intensify competition among microbes [73]. The sudden availability of nutrients could favor fast-growing opportunists, which outcompete others and disrupt mutualistic or cooperative interactions, thereby altering community assembly dynamics [74, 75]. The contrasting effects of prophage induction on bacterial interaction networks in rhizosphere and bulk soil may be due to differing environmental conditions and bacterial phenotypes present in these compartments (Fig.  6). Members of bacterial communities live together and are closely related with each other in the rhizosphere, where their performance and interactions are directly influenced by plant roots [76]. Prophage induction can radically transform bacterial activities and relationships among different members by converting their organizing forms, e.g., deteriorating biofilms [77].

### Viral activity contributes to nutrient turnover and soybean root development

Phages contribute to biogeochemical cycling through AMGs [4] and lysis [2], which may redirect essential elements such as carbon, nitrogen, and phosphorous toward other members of the ecosystem, including bacteria, archaea, fungi, fauna, and plants. Tong *et al*. [8] demonstrated that viral lysis can alleviate microbial nutrient limitations and stimulate the re-utilization of lysate-derived DOC, but did not relate this to the effects of lysogeny-to-lysis switching. Heffner *et al*. [6] reported evidence of a potential mitomycin C-induced viral shunt in landfill cover soil, indicated by an increase in VLP abundance and soluble nutrients. Here, we demonstrated that mitomycin C induction of prophages stimulated microbial activities and nutrient turnover, specifically increasing microbial growth rate (*P* > .05) and organic matter transformation and improving soil nutrient conditions (Fig.  5B and Fig.  3A−C). All these processes were consistently associated with a sharp increase in VLP abundance, a potential increase in viral diversity, and extensive bacterial community turnover. This is consistent with the notion that the viral lysis, particularly the induced lytic activities in lysogenic phages, released nutrients from lysed cells. The flux of nutrients and the selective reduction in populations of susceptible host bacteria restructured the plant-bacteria relationship. Viral lysates contain diverse labile dissolved organic matter that can be quickly utilized by bacterial communities, accelerating microbial activities and nutrient transformations [18, 78]. Viral lytic reproduction also releases various endoenzymes into the environment and increases organic matter decomposition [3, 79]. The increased microbial activities (driven by viral lysis) along with elevated DOC and nitrate-N levels observed in this study (Fig.  3A−C) suggest enhanced organic matter decomposition under prophage induction.

The significantly lower SOC content observed in the prophage induction treatment than in the control treatment indicates that microbial decomposition of organic matter escalated under prophage induction discharging more plant nutrients (Supplementary Fig. S3B). Microbial metabolism and necromass exert instrumental effects in transforming organic compounds and restructure the SOC pool [80]. Intriguingly, the microbial (both bacterial and fungal) necromass content was significantly lower under prophage induction (*P* < .001 for bacterial necromass; *P* < .05 for fungal necromass) (Fig.  7C and D). This reduction in microbial necromass content may be attributed to the elevated microbial activities, particularly of faster growing bacteria, which likely promote reutilization of the necromass carbon. The accelerated decomposition of and subsequent use of necromass by the active microbial community may explain the lower necromass levels despite the overall increase in microbial processes. It is also possible that the microbial necromass generated by the chemical-induced lysis is more readily available for uptake by plants or other microbes [81]. The organic carbon to reactive nitrogen stoichiometry can also further nitrate accrual [82]. The simultaneous decline in N_₂_O emissions and increase in soil nitrate under the prophage-induction treatment is best interpreted as an indirect, microbially mediated outcome of prophage activation rather than as a direct chemical inhibition by mitomycin C. At the applied concentration (0.5 μg·g^−1^ soil), mitomycin C is strongly adsorbed to soil particles and unlikely to reach levels that inhibit denitrification enzymes. Instead, prophage induction reshaped nitrogen-transforming guilds in ways consistent with enhanced nitrification and suppressed denitrification. Specifically, taxa involved in nitrification and nitrogen mineralization, including *Nitrososphaeraceae* (ammonia-oxidizing archaea), *Nitrospira* (nitrite oxidizers), and *Burkholderia–Caballeronia–Paraburkholderia* and *Lysobacter* (decomposers and potential diazotrophs) increased markedly under induction. In contrast, major denitrifiers such as *Methylotenera*, which link methanol oxidation to N_₂_O production [83, 84], and *Saccharimonadales*, which can reduce nitrate to gaseous forms [85], declined significantly (Fig.  4D and F). This guild reorganization, combined with prophage-mediated lysis and altered C:N and redox balance, likely reduced the completeness of the denitrification pathway, limiting N_₂_O formation while favoring nitrate accumulation.

The soil nutrient conditions have a major role in driving rhizobacterial community differentiation and plant-bacteria interactions [86]. The observed enrichment of fast-growing bacterial groups in both rhizosphere and bulk soil compartments under prophage induction was consistent with the elevated nutrient conditions (Fig.  5B). We demonstrated that the viral-induced bacterial activities and shifts in the rhizobacterial community structure ultimately benefited plant growth, specifically decreasing the root diameter and increasing root net length and volume (Fig.  3F, G, and  D). The results suggest that the plasticity and penetration ability of soybean roots were enhanced thereby increasing plant acquisition of resources under prophage induction. Additionally, the modified root phenotypes could influence the structure and properties of the adjacent soils contributing to the SOC turnover [87].

To determine whether mitomycin C directly affects plant root growth independent of microbial mediation, we conducted a supplementary assay using *A. thaliana* (ecotype Col-0) seedlings grown under sterile conditions. It is important to note that this assay differs fundamentally from the soybean pot experiment in both plant species and measurement scale. The soybean pot experiment quantified total root system architecture (including primary, secondary, and lateral roots) after 35 days of growth in soil, resulting in total lengths measured in the hundreds of centimeters. By contrast, the sterile *Arabidopsis* assay measured only primary root elongation of seedlings in agar medium, with values in the millimeter range. These two datasets are therefore not directly comparable in absolute magnitude. The soybean pot experiment provides the most representative data for our central conclusions, as it integrates plant–soil–microbe–phage interactions in an ecologically relevant context. The sterile *Arabidopsis* assay serves as a complementary control to test for potential direct phytotoxicity of mitomycin C in the absence of microbes. The finding that mitomycin C did not inhibit primary root elongation under sterile conditions supports the conclusion that the enhanced soybean root development observed in the pot experiment is primarily mediated by microbially driven processes following prophage induction, rather than direct chemical effects of mitomycin C on plants.

Although our findings support prophage induction as a key driver of the observed shifts in rhizosphere microbial composition, nutrient cycling, and plant development, we acknowledge that mitomycin C may also exert off-target effects on soil microbes beyond its role as a prophage inducer (see Supplementary materials, Section S3). While our validation experiments indicate that the concentration used here selectively induces prophages without broadly affecting non-lysogenic bacteria or plant growth, we cannot fully exclude indirect effects mediated by stress responses or altered microbial metabolism. Therefore, we interpret our results as reflecting the combined influence of prophage activation and potential mitomycin C-induced microbial dynamics. Importantly, the use of synthetic microbial communities (SynComs) represents a promising approach for future studies to disentangle the specific contributions of prophage induction under controlled conditions. Such systems will facilitate deeper mechanistic understanding of prophage-driven processes within plant–soil ecosystems and further clarify the contribution of phage-mediated nutrient release to plant performance and soil microbial dynamics.

## Conclusion

Collectively, our results showed that prophage induction, leading to significant viral production, contributes to nutrient cycling and substantially transforms bacterial communities. Prophage induction stimulated the enrichment of fast-growing bacteria in the rhizosphere. The elevated nutrient levels and increased microbial activities, driven by the lysogeny-to-lysis switch, jointly stimulated soybean root development. This study provides innovative insights into how rhizobacterial community differentiation and plant-microbe interactions are driven by prophage induction-caused intensified viral production. Future efforts may include the following aspects. First, we might examine whether lysogens (bacteria with prophages) have competitive advantages over non-lysogens and elucidate how prophage induction influences bacterial morphology and their colonization on the root surface. Second, we might clarify the functions of AMGs in prophages with experiments. Third, we would like to explore the effects of extracellular phages and prophages on rhizobacterial community assembly and function. These investigations can gain more accurate and quantitative results of phages influencing rhizosphere microbiome structure and functionality. We anticipate that this work could evoke more interest in exploring the functions of phages in rhizosphere processes for a better understanding of plant-microbe-phage interactions.

## Supplementary Material

Data_S1_ycaf203

Data_S2_ycaf203

Supplementary_materials_ycaf203

## Data Availability

All sequencing data supporting this study are publicly available in the NCBI Sequence Read Archive (SRA). The raw bacterial 16S rRNA gene sequences can be accessed under BioProject PRJNA1148935, and raw virome datasets can be accessed under BioProject PRJNA1187349. Remaining data analyzed during this study are included in this published article and its supplementary information and data files.
