# Supplementary material for: Prophage induction drives soybean rhizobacterial community differentiation and nutrient cycling benefiting root development
Source: ISME Commun. 2025 Nov 6;5(1):ycaf203. doi: 10.1093/ismeco/ycaf203 (PMC12645843; doi:10.1093/ismeco/ycaf203)
Supplement: Supplementary_materials_ycaf203 [file supplementary_materials_ycaf203.pdf]

Supplementary Information

Prophage induction drives soybean rhizobacterial community differentiation and nutrient cycling benefiting root development

Running title: Prophage induction reshapes rhizosphere

Yujun Zhong[1,2#], Yingyue Zhang[1,3#], José Luis López Arcondo[4,5], Ruoyi Xu[1,2], Mark Radosevich[6], Jeffery L. Dangl[7,8], Bas E. Dutilh[4, 9], and Xiaolong Liang[1,2]*

[1] CAS Key Laboratory of Forest Ecology and Silviculture, Institute of Applied Ecology, Chinese Academy of Sciences, Shenyang, China

[2] University of Chinese Academy of Sciences, Beijing, China

[3] Institute of Carbon Neutrality Technology and Policy, Shenyang University, Shenyang, China

[4] Institute of Biodiversity, Faculty of Biological Sciences, Cluster of Excellence Balance of the Microverse, Friedrich Schiller University Jena, Jena, Germany

[5] Instituto Andino Patagónico de Tecnologías Biológicas y Geoambientales, Bariloche, Rio Negro, Argentina

[6] Department of Biosystems Engineering and Soil Science, The University of Tennessee, Knoxville, TN, U.S.A

[7] Department of Biology, University of North Carolina at Chapel Hill, Chapel Hill, NC, U.S.A.

[8] Howard Hughes Medical Institute, University of North Carolina at Chapel Hill, Chapel Hill, NC, U.S.A.

[9] Theoretical Biology and Bioinformatics, Department of Biology, Science for Life, Utrecht University, Utrecht, The Netherlands

# Co-first authors

*Corresponding author: Xiaolong Liang

Mailing address: Institute of Applied Ecology, Chinese Academy of Sciences, No. 72 Wenhua Road, Shenyang, Liaoning Province 110016, China

†Current affiliation: Howard Hughes Medical Institute, Department of Biology, University of North Carolina at Chapel Hill, Chapel Hill, NC, USA

Email: liangxlucas@outlook.com

Section S1

Materials and Methods

Prophage identification in tested bacterial strains

To investigate strain-level responses to mitomycin C, we selected a panel of eight bacterial isolates (i.e., *Stutzerimonas stutzeri* H46, *Chitinophaga* sp. EN-1, *Arthrobacter* sp. OA17, *Rhodococcus* sp. 1A6, *Bacillus cereus* TJ-25, *Priestia megaterium* R-6, *Pseudomonas aeruginosa* FYS20, and *Klebsiella* sp. FX20-3) mostly obtained from the same black soil used in the soybean pot experiment. This panel included both putative lysogenic and non-lysogenic strains. A laboratory reference strain, *Escherichia coli* DH5α, was also included as a non-lysogenic negative control in prophage induction assays. Whole-genome sequencing was performed for each isolate, and prophage regions were predicted using the PhiSpy software package (version 4.2.21, default parameters). This tool integrates multiple genomic features, including gene content, GC composition, and hallmark phage structural and regulatory genes, to delineate prophage boundaries with high accuracy. Strains containing intact prophage elements with identifiable structural and lytic genes were classified as lysogenic, whereas isolates lacking such regions or containing only degraded remnants were considered non-lysogenic. To validate these predictions, induction assays with a positive control treatment (1.0 μg/mL mitomycin C) were used to confirm the release of virus-like particles (VLPs) in lysogenic strains, while no VLPs were detected from prophage-free isolates.

Bacterial growth and viability assays under mitomycin C treatment

To evaluate the influence of mitomycin C on bacterial growth, individual strains were first revived from frozen glycerol stocks in Luria–Bertani (LB) broth (10 g/L tryptone, 5 g/L yeast extract, 10 g/L NaCl, pH 7.0). Cultures were inoculated at 2% (v/v) and incubated at 30 °C with shaking at 160 rpm for 24 h. Log-phase cells were then transferred into fresh LB broth containing different mitomycin C concentrations (0, 0.1, 0.5, and 1.0 μg/mL). The culture without mitomycin C served as the untreated control.

To further examine the potential cytotoxic effects of mitomycin C and to distinguish prophage induction from general antimicrobial activity, additional viability assays were conducted under nutrient-free conditions. Mid-exponential cultures were harvested by centrifugation at 8,000 × g for 3 min, washed twice in potassium citrate–phosphate buffer (1.44 g/L $Na_2HPO_4 \cdot 7H_2O$, 0.24 g/L KH_2PO_4, 10 g/L potassium citrate, pH 7.0), and resuspended to a density of approximately 2×10^8 cells/mL. Aliquots were exposed to 0, 0.5, or 1 µg/mL mitomycin C and incubated at 25 °C with shaking at 160 rpm in the dark (to minimize mitomycin C photodegradation) for 18 h. Following exposure, cells were collected by centrifugation (6,000 × g, 5 min), washed twice to remove residual mitomycin C, and resuspended in fresh buffer.

Cell viability was determined by colony-forming unit (CFU) enumeration. Serial 10-fold dilutions of each suspension were spotted (5 µL, in triplicate) onto LB agar plates and incubated at 37 °C for 24 h. Only plates containing 30–300 colonies were used to calculate CFU counts, which were expressed as CFU/mL. Mortality rates were calculated relative to the untreated (0 µg/mL mitomycin C) control. Mortality rates were calculated as:

$$Mortality\ rate = \frac{N_0 - N_{MitC}}{N_0}$$

where N_0 represents the viable cell count in the control (0 µg/mL MitC) and N_{MitC} represents the viable cell count in the MitC-treated cultures (at 0.5 or 1 µg/mL MitC) after 18 h cultivation. This ensured that the 0 µg/mL control was always explicitly included as the baseline for comparison. This approach provided a robust quantification of surviving cells and allowed discrimination between general cytotoxicity and prophage induction-mediated lysis.

Preparation of soil- and water-derived microbial consortia

To complement the single-strain induction assays, we established mixed microbial consortia to evaluate mitomycin C effects on community-level growth dynamics. The soil consortium was prepared from the same black soil used in the soybean pot experiment, ensuring direct relevance to the main study system. Approximately 50 g of

soil was homogenized in sterile phosphate-buffered saline (PBS), centrifuged at 500 × g for 5 min to remove debris, and the supernatant was passed through 5 μm filters to collect microbial cells while excluding larger eukaryotes. The water consortium was collected from a freshwater pond at the Suburb Experimental Station of the Institute of Applied Ecology, CAS, Shenyang, China (41°91′N, 123°59′E), processed identically to the soil samples, and used as an ecological contrast. Aliquots of each inoculum (1% v/v) were cultured in 50 mL R2A broth in 250 mL Erlenmeyer flasks under three conditions: 0 μg/mL mitomycin C (control), 0.5 μg/mL mitomycin C (soil-equivalent dose), and 1 μg/mL mitomycin C (positive induction control). Growth was monitored by optical density at 595 nm (OD_{595}) using a UV–Vis spectrophotometer over 48 h at 28 °C, 200 rpm.

Section S2

Results and discussion

The comparison between the soil-based soybean experiment and the axenic *Arabidopsis* assay further underscores the context-dependent nature of MitC effects. In the pot experiment, soybean root traits were quantified at the whole-system level, with total lengths including primary and fine lateral roots reaching hundreds of centimeters. By contrast, the axenic assay measured only primary root elongation of seedlings grown on agar plates, yielding values in millimeters. These methodological and biological differences explain the discrepancy in scale between Fig. 3G and Fig. S4. Importantly, the pot experiment is the more representative system for our conclusions, as it integrates the full suite of plant–soil–microbe–phage interactions. The axenic assay provides complementary evidence that MitC at the applied soil-equivalent dose (0.5 μg/g) does not exert direct phytotoxic effects on plant roots. Taken together with our strain-level and consortia experiments, these results support the conclusion that the observed stimulation of soybean root development arises primarily from prophage-mediated microbial processes rather than broad-spectrum or plant-directed toxicity of MitC.

Section S3

Results and discussion

Community-level responses of microbial consortia to MitC

To complement the single-strain assays, we evaluated the growth of mixed microbial consortia derived from two sources: (i) the same black soil used in the soybean pot experiment and (ii) a freshwater pond community used as an ecological contrast. Across 48 h of incubation, both consortia exhibited typical logistic growth curves, and no significant differences in OD_{595} were observed between MitC-treated (0.5 μg/mL) and untreated controls (Fig. S1B). At the higher concentration (1.0 μg/mL), a modest reduction in final biomass was detected, consistent with the broad-spectrum cytotoxicity of MitC at elevated doses. The absence of an effect at the soil-equivalent dose suggests that prophage induction likely occurred in only a subset of lysogenic taxa, and that these selective lysis events were insufficient to alter bulk biomass in well-mixed liquid cultures. These results indicate that the ecological consequences of prophage induction are not apparent when measured solely as community-level biomass in liquid medium but are amplified in structured soil environments where localized lysis and nutrient release can reshape microbial interactions.

The microbial consortia assays provide additional insight into the selectivity of MitC-induced prophage activation. At the soil-equivalent concentration (0.5 μg/mL), no significant differences in bulk biomass were observed between treated and untreated consortia, despite evidence from strain-level assays and soil experiments that prophage induction occurred. This apparent discrepancy likely reflects the difference between well-mixed liquid cultures and structured soil environments. In liquid medium, OD-based growth integrates all taxa and can obscure the selective lysis of lysogenic subpopulations, as non-lysogenic members continue to grow and maintain overall biomass. By contrast, in the soil matrix, prophage induction in localized host populations can release labile nutrients in microsites, intensifying microbial competition and accelerating community turnover even without detectable changes in total biomass. Together, these assays support the conclusion that at the

applied dose, MitC does not act as a broad-spectrum antimicrobial but instead triggers prophage induction in a subset of lysogens, with ecological consequences that are strongly dependent on environmental context.

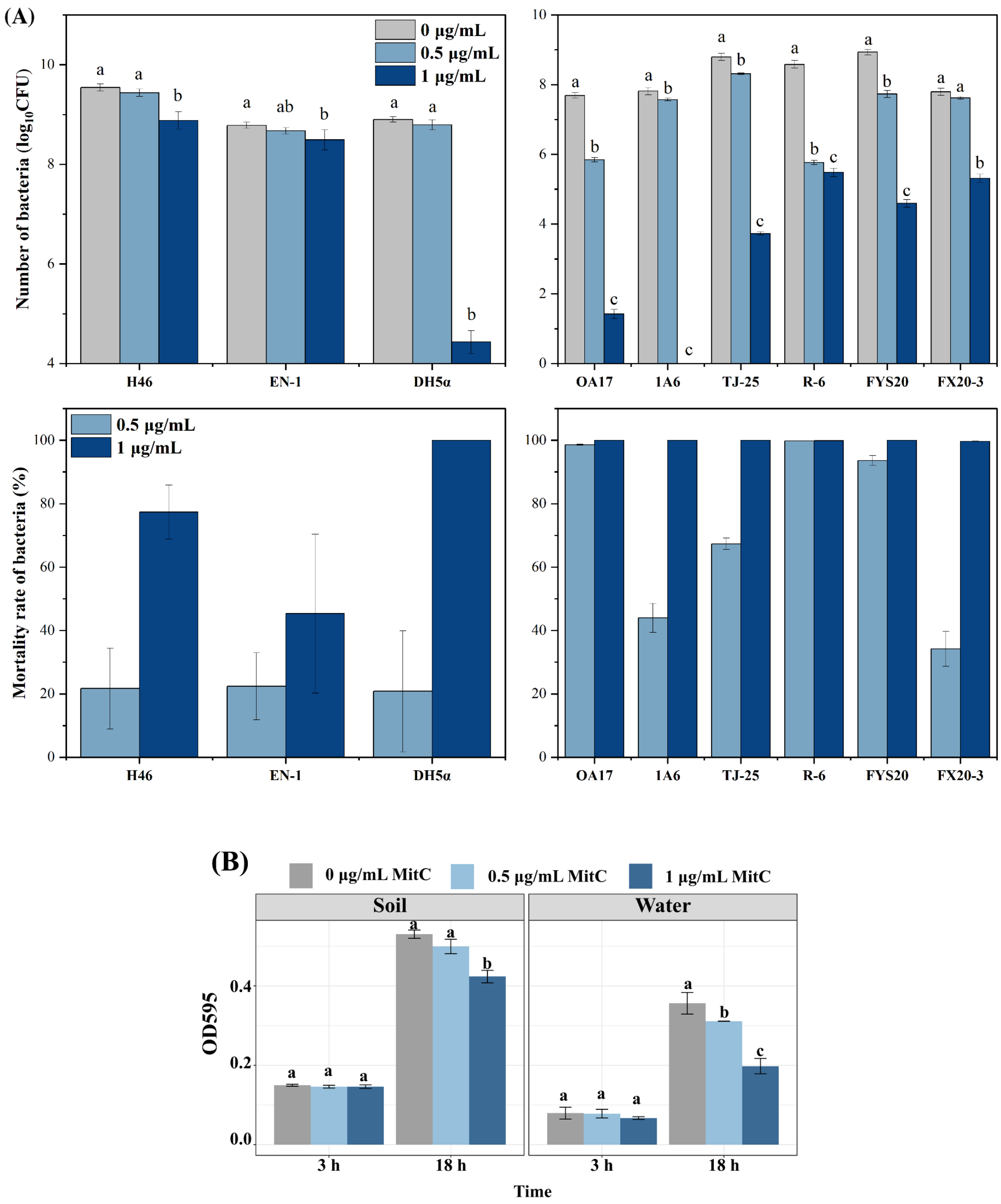

Fig. S1 Direct effects of mitomycin C on bacteria. (A) Mortality rates of bacterial populations in induction assays using nine bacterial strains: *Stutzerimonas stutzeri* H46 (non-lysogen), *Chitinophaga* sp. EN-1 (non-lysogen), *Escherichia coli* DH5α (non-lysogen), *Arthrobacter* sp. OA17 (lysogen), *Rhodococcus* sp. 1A6(lysogen), *Bacillus cereus* TJ-25 (lysogen), *Priestia megaterium* R-6 (lysogen), *Pseudomonas aeruginosa* FYS20 (lysogen), and *Klebsiella* sp. FX20-3 (lysogen). Each strain was incubated in 1× phosphate-buffered saline (PBS) supplemented with 0, 0.5, or 1 μg/mL mitomycin C for 18 hours. (B) Effects of mitomycin C on complex environmental bacterial

consortia derived from soil and water samples. The consortia were cultured in minimal medium supplemented with 0, 0.5, or 1 μg/mL mitomycin C. Bacterial growth (OD595) was monitored over an 18-hour period. Each data point represents mean of triplicate biological replicates; error bars indicate standard deviation.

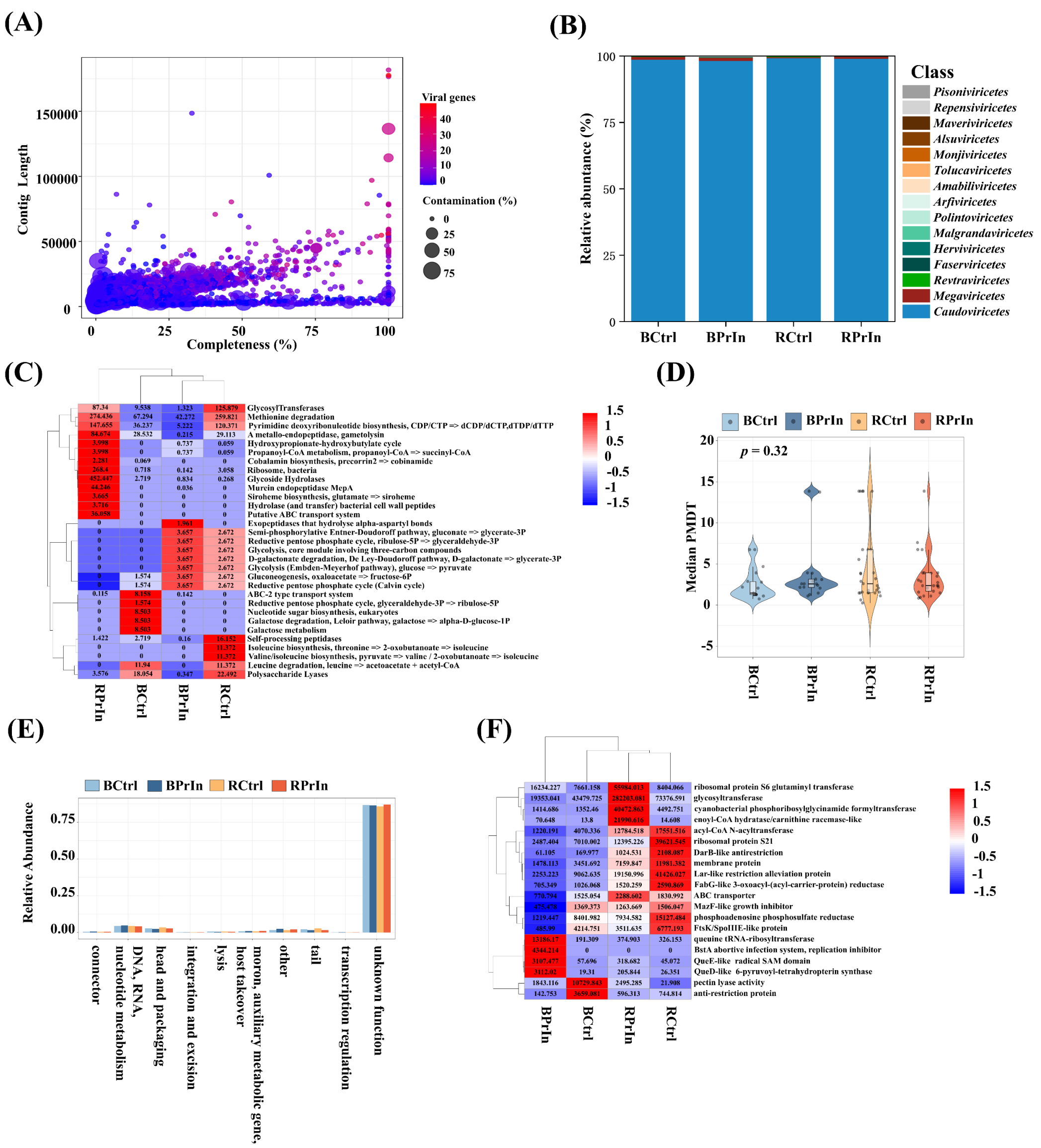

Fig. S2 (A) CheckV quality assessment was performed on the predicted viral contigs. Completeness, contamination, and viral gene content are displayed from the CheckV results. (B) Reads relative abundances to viral clusters. Reads from the four samples (BCtrl, BPrIn, RCtrl, and RPrIn) were mapped to contigs and clusters that could be assigned up to class level based on geNomad annotations are represented. (C) Heatmap of relative abundances of predicted AMG modules. Counts were summed per AMG module as per DRAM-v classification, normalized by sample sequencing read counts and expressed per million reads. (D) Median predicted maximum growth rates of predicted hosts in viromes. Kruskall Wallis test resulted in no significant differences between treatments. (E) Normalized abundances of functional categories in viral

contigs from each sample. (F) Normalized reads mapping to genes in category "Moron, auxiliary metabolic genes, and host take-over" in pharokka annotations. All representative contigs were analyzed. Counts were summed per functional annotation, normalized by sample sequencing read counts and expressed per million reads. Treatments are: BCtrl: bulk soil of control; BPrIn: bulk soil of prophage induction treatment; RCtrl: rhizosphere of control; RPrIn: rhizosphere of prophage induction treatment.

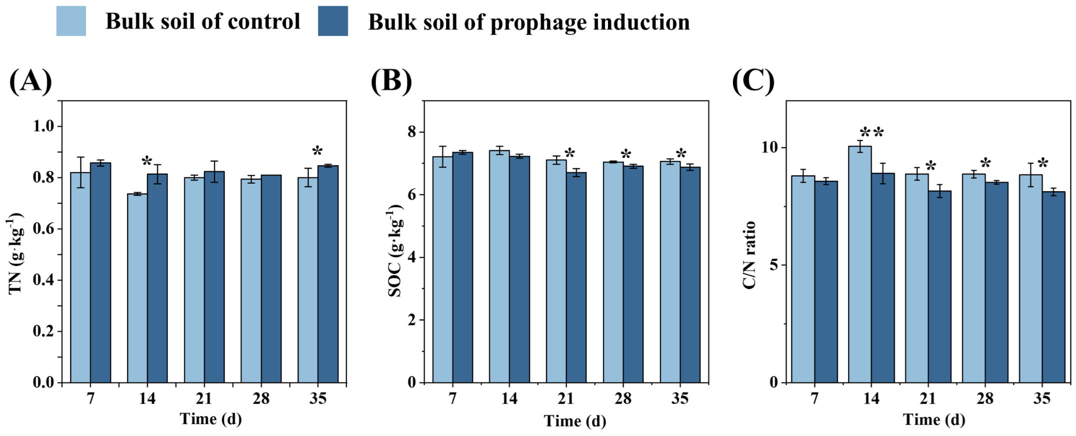

Fig. S3 The content of total nitrogen (TN) (A) soil organic C (SOC) (B) and carbon/nitrogen ratio (C/N ratio) (C) in bulk soils. Statistical differences between different treatments were determined according to Student's *t*-test, and the significant comparison was labeled accordingly (*: $p < 0.05$; **: $p < 0.01$; ***: $p < 0.001$). Treatments are: BCtrl: bulk soil of control; BPrIn: bulk soil of prophage induction treatment.

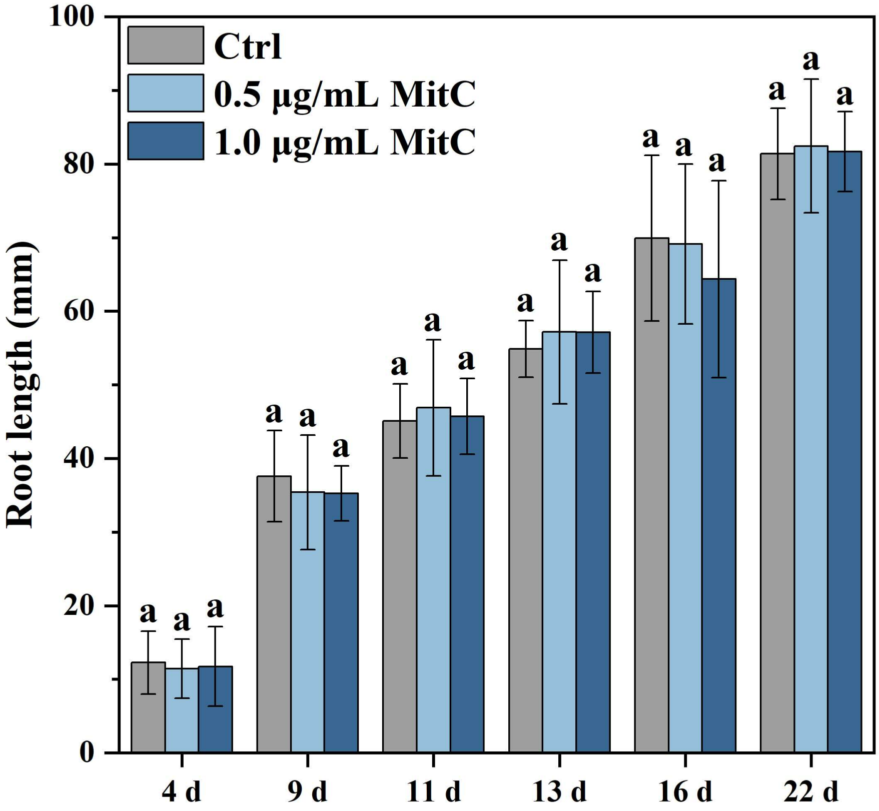

Fig. S4 Effects of mitomycin C (MitC) on primary root elongation of *Arabidopsis thaliana* (Columbia-0 ecotype) seedlings under sterile conditions. Seeds were germinated and cultivated on MS agar medium supplemented with 0, 0.1, or 1.0 μg/mL MitC. Primary root length was measured at 4, 9, 11, 13, 16, and 22 days after germination. Values represent means ± standard errors (n = 5). Error bars indicate standard deviation. Statistical differences between groups were assessed using analysis of variance (ANOVA) followed by post hoc testing (Duncan's multiple range test). Groups labeled with different letters (a, b, c) are significantly different ($p < 0.05$).

Note: Root lengths in this assay are expressed in millimeters and reflect the primary root growth of *Arabidopsis* seedlings in sterile Murashige and Skoog (MS) agar medium. These values are not directly comparable to those in Fig. 3G, which measured the total length of highly branched soybean root systems grown for 35 days in soil, expressed in centimeters.

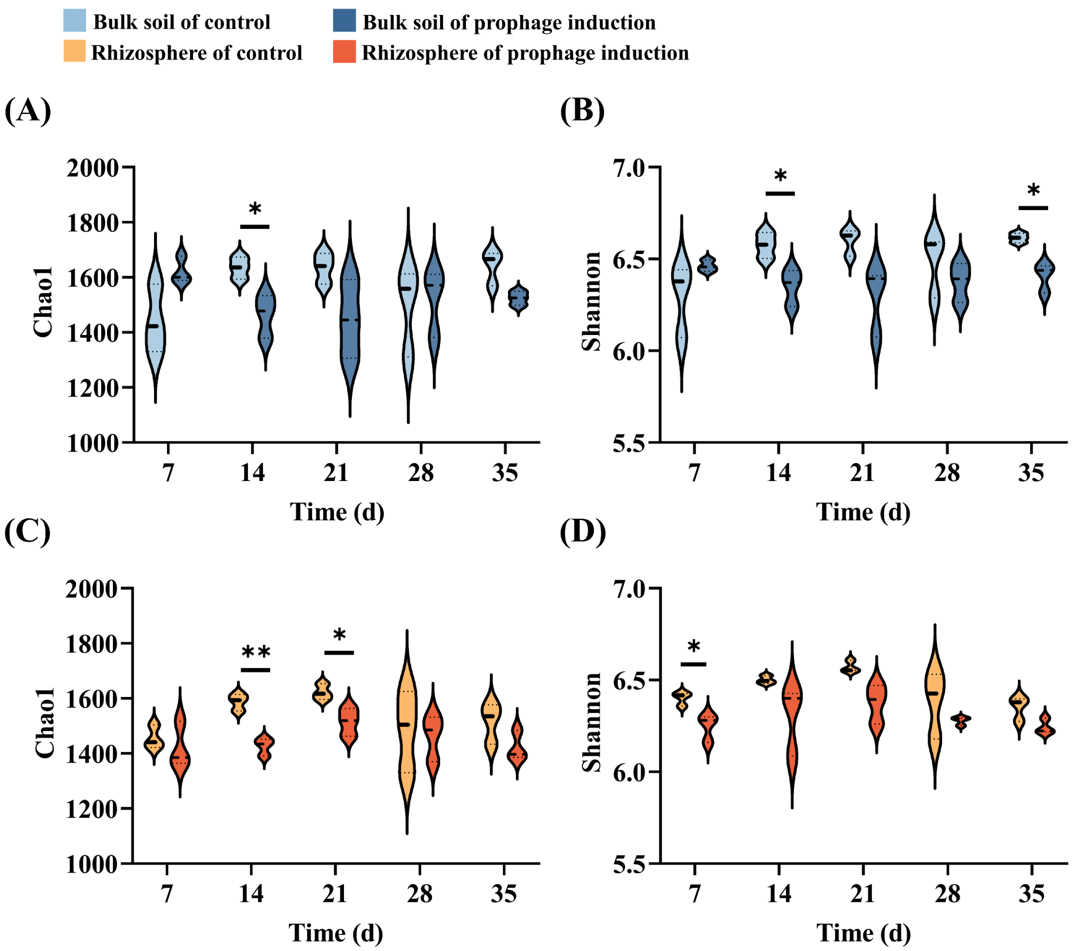

Fig. S5 Bacterial community species richness and diversity in bulk soil and the rhizosphere. (A) Chao1 richness index of bacterial communities in the rhizosphere. (B) Shannon diversity index of bacterial communities in the rhizosphere. (C) Chao1 richness index of bacterial communities in bulk soils. (D) Shannon diversity index of bacterial communities in bulk soils. Statistical differences between different treatments were determined according to Student's *t*-test, and the statistical significance is labeled accordingly (*: $p < 0.05$; **: $p < 0.01$; ***: $p < 0.001$).

Treatments are: BCtrl: bulk soil of control; BPrIn: bulk soil of prophage induction treatment; RCtrl: rhizosphere of control; RPrIn: rhizosphere of prophage induction treatment.

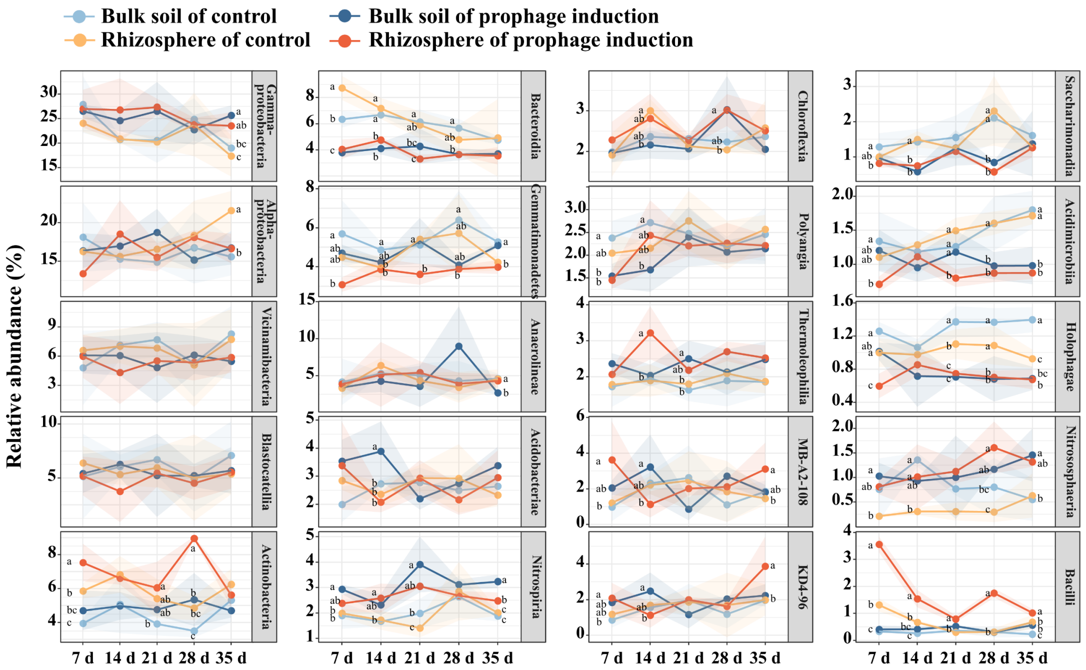

Fig. S6 Taxonomic composition of bacterial communities at the class level during incubation. Each point represents the mean relative abundance of triplicate samples; shaded regions indicate the mean ± standard deviation. Statistical comparisons among groups were performed using ANOVA followed by Duncan's multiple range test, and significant differences ($p < 0.05$) are denoted by different letters (a, b, c). The ten most abundant classes across all samples were Gammaproteobacteria, Alphaproteobacteria, Vicinamibacteria (Acidobacteriota), Blastocatellia (Acidobacteriota), Actinobacteria, Bacteroidia (Bacteroidota), Gemmatimonadetes, Anaerolineae (Chloroflexota), and Nitrospiria (Nitrospirota). Relative abundances of Vicinamibacteria, Bacteroidia, Gemmatimonadetes, Polyangia (Myxococcota), Saccharimonadia (Patescibacteria), Acidimicrobiia (Actinobacteriota), and Holophagae (Acidobacteriota) declined under prophage induction, whereas Gammaproteobacteria, Nitrospiria, Thermoleophilia (Actinobacteriota), Nitrososphaeria (Nitrososphaerota), and Bacilli (Bacillota) were specifically enriched in rhizosphere soils exposed to induction.

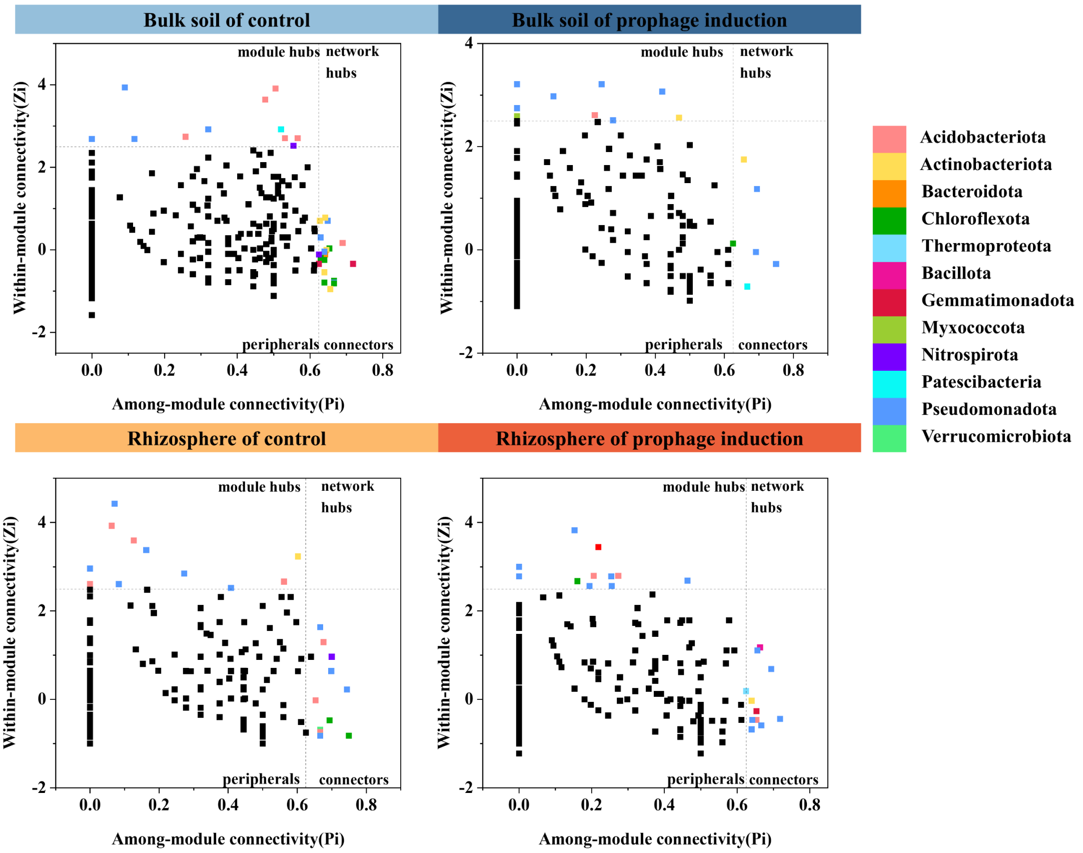

Fig. S7 Node connectivity in molecular ecological networks of soil bacterial communities. Each square represents a taxonomic node, with the size of the square proportional to its connectivity within the network. Colored squares indicate potential keystone taxa, and colors correspond to their taxonomic assignments at the class level. Keystone species were identified based on high within-module and among-module connectivity, highlighting their central roles in structuring bacterial community interactions under different treatments.

Table S1 Number of high-quality virome enriched metagenomic reads.

Treatments are: BCtrl: bulk soil of control; BPrIn: bulk soil of prophage induction treatment; RCtrl: rhizosphere of control; RPrIn: rhizosphere of prophage induction treatment.

Treatment	Reads
BCtrl	72448458
BPrIn	112669552
RCtrl	78040188
RPrIn	67357708

Table S2 Richness and Shannon diversity index of virome in rhizosphere and bulk soils.

Treatments are: BCtrl: bulk soil of control; BPrIn: bulk soil of prophage induction treatment; RCtrl: rhizosphere of control; RPrIn: rhizosphere of prophage induction treatment.

Treatment	Richness	Shannon
BCtrl	10118	11.56
BPrIn	8995	11.17
RCtrl	8703	11.20
RPrIn	11553	11.33

Table S3 Summary of functional annotations using pharokka.

Treatments are: BCtrl: bulk soil of control; BPrIn: bulk soil of prophage induction treatment; RCtrl: rhizosphere of control; RPrIn: rhizosphere of prophage induction treatment.

	**BCtrl**	**BPrIn**	**RCtrl**	**RPrIn**
unknown function	62097	45903	64084	75427
DNA, RNA and nucleotide metabolism	2453	1920	2687	3068
head and packaging	1737	1221	2065	2368
other	1341	956	1554	1754
tail	761	544	1072	1262
tRNAs	1005	523	814	1018
moron, auxiliary metabolic gene and host takeover	672	489	715	814
integration and excision	446	398	445	592
connector	254	193	330	397
lysis	223	163	288	285
transcription regulation	159	139	194	206
CRISPRs	7	20	12	6
tmRNAs	5	10	11	18
VFDB_Virulence_Factors	1	1	6	2
CARD_AMR_Genes	1	0	0	0

Table S4 Differential abundance analysis using MaAsLin2 in rhizosphere at phylum-level.

Treatments are: BCtrl: bulk soil of control; BPrIn: bulk soil of prophage induction treatment; RCtrl: rhizosphere of control; RPrIn: rhizosphere of prophage induction treatment.

Taxa (phylum-level)	value	coef	stderr	Pvalue	AdjPvalue	Significance
Thermoproteota	RPrIn	1.90	0.30	9.3E-07	3.1E-05	***
Bacillota	RPrIn	1.37	0.29	7.0E-05	5.8E-04	***
Nitrospirota	RPrIn	0.45	0.13	1.3E-03	5.4E-03	**
Actinobacteriota	RPrIn	0.21	0.06	2.3E-03	7.5E-03	**
Gemmatimonadota	RPrIn	-0.35	0.09	6.5E-04	3.1E-03	**
Methylomirabilota	RPrIn	-0.58	0.18	3.6E-03	1.1E-02	*
Bacteroidota	RPrIn	-0.64	0.15	2.7E-04	1.8E-03	**
Patescibacteria	RPrIn	-0.68	0.17	4.6E-04	2.5E-03	**
Fibrobacterota	RPrIn	-1.85	0.55	2.1E-03	7.5E-03	**
Bdellovibrionota	RPrIn	-2.57	0.51	2.3E-05	2.6E-04	***
Cyanobacteriota	RPrIn	-2.76	0.53	1.7E-05	2.6E-04	***

Table S5 The properties of bacterial co-abundance networks in rhizosphere and bulk soils.

Treatments are: BCtrl: bulk soil of control; BPrIn: bulk soil of prophage induction treatment; RCtrl: rhizosphere of control; RPrIn: rhizosphere of prophage induction treatment.

	Keystone species	Negative interaction	Positive interaction	Nodes	Edges
BCtrl	33	34.17%	65.83%	437	1586
BPrIn	17	31.60%	68.40%	364	864
RCtrl	25	27.14%	72.86%	405	910
RPrIn	24	25.15%	74.85%	386	1010
